# Differential cytotoxicity induced by the Titanium(IV)Salan complex Tc52 in G2-phase independent of DNA damage

**DOI:** 10.1186/s12885-016-2538-0

**Published:** 2016-07-13

**Authors:** Theresa Pesch, Harald Schuhwerk, Philippe Wyrsch, Timo Immel, Wilhelm Dirks, Alexander Bürkle, Thomas Huhn, Sascha Beneke

**Affiliations:** University of Zurich, Institute of Veterinary Pharmacology and Toxicology, 8057 Zurich, Switzerland; University of Konstanz, Molecular Toxicology Group, 78457 Konstanz, Germany; Department of Chemistry, University of Konstanz, 78457 Konstanz, Germany; Leibniz Institute DSMZ, Molecular Biology Group, 38124 Braunschweig, Germany; Present address: Leibniz Institute for Age Research: FLI, Beutenbergstr. 11, 07745 Jena, Germany; Present address: Department of Biology, University of Konstanz, Ecotoxicology Group, 78457 Konstanz, Germany; Present address: Lanxess, 41539 Dormagen, Germany

**Keywords:** Titanium(IV)salan complex, Apoptosis, Senescence, Cell-cycle, Tumorigenicity

## Abstract

**Background:**

Chemotherapy is one of the major treatment modalities for cancer. Metal-based compounds such as derivatives of cisplatin are in the front line of therapy against a subset of cancers, but their use is restricted by severe side-effects and the induction of resistance in treated tumors. Subsequent research focused on development of cytotoxic metal-complexes without cross-resistance to cisplatin and reduced side-effects. This led to the discovery of first-generation titanium(IV)salan complexes, which reached clinical trials but lacked efficacy. New-generation titanium (IV)salan-complexes show promising anti-tumor activity in mice, but their molecular mechanism of cytotoxicity is completely unknown.

**Methods:**

Four different human cell lines were analyzed in their responses to a toxic (Tc52) and a structurally highly related but non-toxic (Tc53) titanium(IV)salan complex. Viability assays were used to reveal a suitable treatment range, flow-cytometry analysis was performed to monitor the impact of dosage and treatment time on cell-cycle distribution and cell death. Potential DNA strand break induction and crosslinking was investigated by immunostaining of damage markers as well as automated fluorometric analysis of DNA unwinding. Changes in nuclear morphology were analyzed by DAPI staining. Acidic beta-galactosidase activity together with morphological changes was monitored to detect cellular senescence. Western blotting was used to analyze induction of pro-apoptotic markers such as activated caspase7 and cleavage of PARP1, and general stress kinase p38.

**Results:**

Here we show that the titanium(IV)salan Tc52 is effective in inducing cell death in the lower micromolar range. Surprisingly, Tc52 does not target DNA contrary to expectations deduced from the reported activity of other titanium complexes. Instead, Tc52 application interferes with progression from G2-phase into mitosis and induces apoptotic cell death in tested tumor cells. Contrarily, human fibroblasts undergo senescence in a time and dose-dependent manner. As deduced from fluorescence studies, the potential cellular target seems to be the cytoskeleton.

**Conclusions:**

In summary, we could demonstrate in four different human cell lines that tumor cells were specifically killed without induction of major cytotoxicity in non-tumorigenic cells. Absence of DNA damaging activity and the cell-cycle block in G2 instead of mitosis makes Tc52 an attractive compound for further investigations in cancer treatment.

**Electronic supplementary material:**

The online version of this article (doi:10.1186/s12885-016-2538-0) contains supplementary material, which is available to authorized users.

## Background

Cancer is the second most frequent cause of death in industrial countries. Treatments to cure the disease range from classical surgery, high-energy irradiation [1–3] or DNA damaging drugs [4–9] and chemicals interfering with DNA repair [10–12] to compounds tackling signaling cascades [13–18] or the cytoskeleton [19–21] and combinations thereof. One success story was the discovery of cisplatin as chemotherapeutic agent [22]. Platinum-compounds still play a role in chemotherapy, but their efficiency is limited to a minor cancer-panel and hampered by severe side-effects and acquired resistance [23]. Current research focuses on other metal-complexes, with little cross-reactivity to cisplatin and reduced side-effects. Several cytotoxic titanium-based complexes have been investigated, with titanocene-dichloride and budotitane reaching clinical trials [24–27]. Unfortunately, both substances display a fast rate of hydrolysis [28], resulting in low efficacy and cancelling of phase-II trials. Titanium(IV)salans displaying much longer half-life in aqueous environments [29] and an antitumor-efficacy comparable to cisplatin [29, 30], but with no cross-resistance [30]. Apoptosis induction by two differently substituted complexes in tumor cell lines was described [29], and efficacy was shown in a mouse tumor-model [31]. Titanium(IV)salans mode of action is unknown, but titanocene-dichloride was enriched in chromatin regions [32], bound to DNA and inhibited DNA-synthesis and topoisomerase II [33]. In order to elucidate the cytotoxic mechanism of titanium(IV)salans, we treated four different human cell lines with the cytotoxic compound Tc52 [29] or the - despite a high degree in structural identity - non-toxic Tc53 [34, 35] (Additional file 1: Figure S1). We could not detect any signs of DNA damage, but cytotoxicity was selective for the tumorigenic cell lines investigated, accompanied by a G2-phase cell-cycle block. Non-tumor cells were spared from death, and fibroblasts underwent senescence. By analyzing different mitosis-targeting toxins together with Tc52, we could show that the putative target is the cytoskeleton.

## Results

### DNA damage is not induced by Tc52

**Fig. 1:**
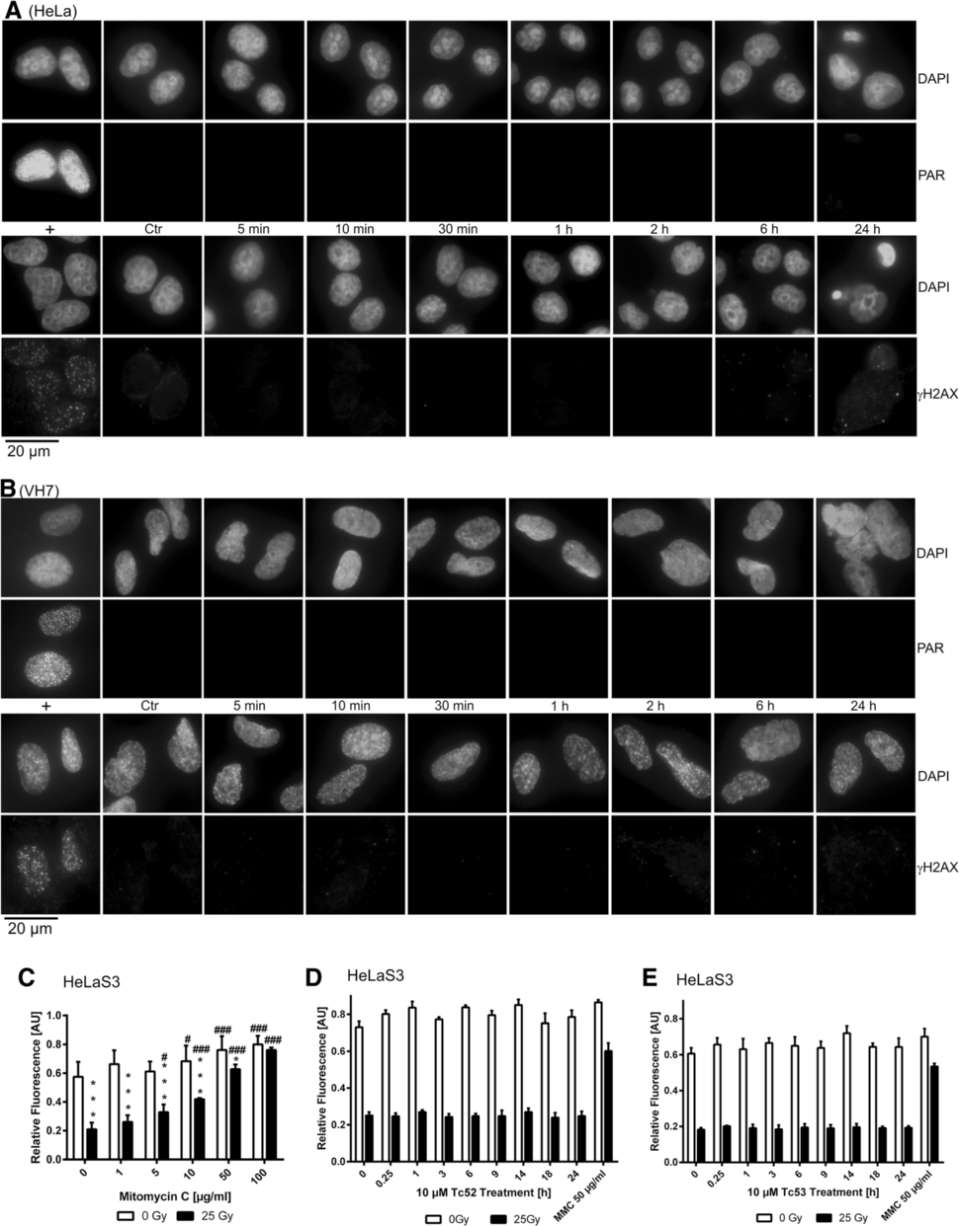
*Tc52 does not induce DNA damage.* **a**: Detection of PAR (upper panel) and γH2AX (lower panel) by immunofluorescence after application of 10 μM Tc52 in HeLa cells. For positive controls (+), cells were incubated with 500 μM H_2_O_2_ for 10 min (PAR detection) and for 6 h (γH2AX detection). Respective upper rows show staining of nuclei (DAPI), lower rows detection of either PAR or γH2AX. Incubation time is depicted between both panels. γH2AX is only evident in apoptotic cells after 24 h. For each independent experiment, at least 100 cells were analyzed in technical duplicates. **b** Detection of PAR (upper panel) and γH2AX (lower panel) by immunofluorescence after application of 10 μM Tc52 in VH7 normal fibroblasts. For positive controls (+), cells were incubated with 500 μM H_2_O_2_ for 30 min (PAR detection) and for 1 h (γH2AX detection). Respective upper rows show staining of nuclei (DAPI), lower rows detection of either PAR or γH2AX. Incubation time is depicted between both panels. For each independent experiment, at least 100 cells were analyzed in technical duplicates. Complete time course of H_2_O_2_ treatment is depicted in Additional Fig. 3a for HeLa cells and Additional Fig. 3b for VH7 fibroblasts. **c**: Dose–response curve for mitomycin C-dependent crosslinking in HeLa. Bars indicate fluorescence signals with (filled bars) or without (open bars) 25 Gy irradiation as detected by FADU. Irradiation reduces, application of mitomycin C increases signals. Asterisks (*) indicate significant difference between irradiated and non-irradiated samples; hashtags (#) describe significance compared to the respective controls (0). *p* < 0.05 = #/*, *p* < 0.01 = ##/**; *p* < 0.001 = ###/***, (two-way ANOVA, Sidak's Multiple Comparison Test). At 100 μM mitomycin C, strong crosslinking prevents drop in signal intensity by irradiation. **d**: Time course of DNA breaks and crosslink detection by FADU after application of 10 μM Tc52 to HeLa cells for the indicated time points. Bars indicate fluorescence signals with (filled bars) or without (open bars) 25 Gy irradiation as detected by FADU. No change in signal intensity over time can be observed. 50 μg/ml mitomycinC (MMC) was applied as positive control. **e**: Time course of DNA breaks and crosslink detection by FADU after application of 10 μM Tc53 to HeLa cells for the indicated time points. Bars indicate fluorescence signals with (filled bars) or without (open bars) 25 Gy irradiation as detected by FADU. No change in signal intensity over time can be observed. 50 μg/ml mitomycinC (MMC) was applied as positive control

We investigated titanium-compounds Tc52 and Tc53 in the well-established HeLa cancer cell line and in normal VH7 fibroblasts. Both cell lines displayed similar sensitivity in viability to Tc52 (EC_50_ and EC_80_ of 6 μM and 10 μM in HeLa, and of 3.5 μM and 10 μM in VH7, respectively), whereas Tc53 had no effect (Additional file 2: Figure S2). This is interesting, as Tc53 has a very similar overall structure and differs from Tc52 only by the exchange of four methyl-groups against tert-butyl groups. Major impact of Tc52 was detected after 48 h incubation, similar to published results [29]. As titanocene-dichloride was reported to interact with DNA [33], we investigated for direct or indirect induction of DNA-damage by Tc52. We monitored for the appearance of DNA-break markers poly(ADP-ribose) (PAR) and phosphorylation of histone H2AX (γH2AX), using H_2_O_2_ as positive control (Additional file 3: Figure S3A/B). Neither Tc52 (Fig. 1a/b) nor Tc53 (data not shown) induced any DNA strand-break marker up to 6 h of continuous treatment. However, after 24 h a very small subset of HeLa cells displayed γH2AX induction, coinciding with the onset of cell death. Patterns of γH2AX formation in western-blots of HeLa cell extracts after Tc52, Tc53 or H_2_O_2_ application mirrored the results from immunofluorescence studies (Additional file 3: Figure S3C). To detect DNA breaks and crosslinks, we employed the sensitive automated FADU and reverseFADU methods [36, 37]. Mitomycin C as crosslinking agent showed the expected dose-dependent fluorescence increase in reverseFADU (Fig. 1c), whereas Tc53 (10 μM) was negative (Fig. 1d). In line with data from immunofluorescence and western-blotting, 10 μM Tc52 neither induced DNA-breaks nor crosslinks (Fig. 1e). In summary, we could not detect any Tc52-dependent DNA breakage or crosslinking activity.

### Tc52 blocks cells in G2-phase

**Fig. 2:**
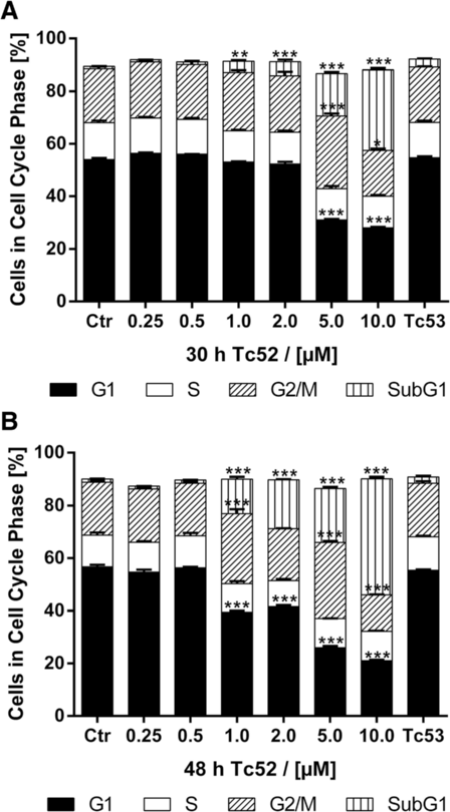
*Dose-dependent changes in cell-cycle profile by Tc52.* **a**: Cell-cycle distribution of HeLa cells after 30 h incubation with increasing concentrations of Tc52. Filled bars: G1; open bars: S; hatched bars: G2/M; vertical line bars: subG1. Dose-dependent increase (30-fold) in subG1 from 1 μM to 10 μM with significant decrease in G1 starting at 5 μM (2-fold), the latter accompanied by accumulation in G2/M (1.5-fold). Tc53 has no impact on cell-cycle distribution. **b**: Cell-cycle distribution of HeLa cells after 48 h incubation with increasing concentrations of Tc52. Filled bars: G1; Pointed bars: S; Hatched bars: G2/M; Open bars: subG1. Dose-dependent increase (44-fold) in subG1 from 1 μM to 10 μM concomitant with significant decrease in G1 (2.7-fold), accompanied by fluctual accumulation in G2/M. Tc53 has no impact on cell-cycle distribution. Treatments were compared to controls and significance was calculated by two-way ANOVA with Dunnett's Multiple Comparison Test. *p* < 0.05 = *, *p* < 0.01 = **, *p* < 0.001 = ***

**Fig. 3:**
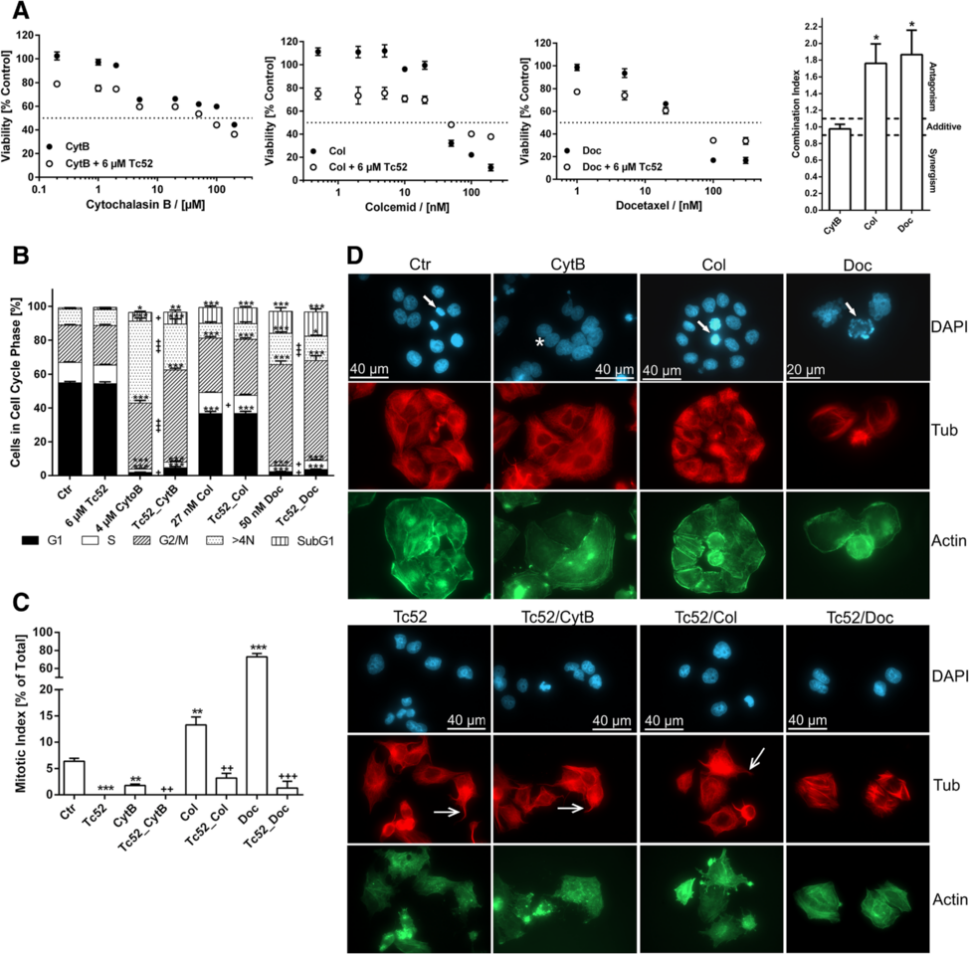
*Tc52 blocks cells in G2 and alters tubulin network.* Treatment of HeLa tumor cells with toxins targeting different steps in M-phase in combination with Tc52. M-phase drugs were administered in increasing concentrations either alone or together with 6 μM Tc52 (EC_50_) for 24 h. **a**: Viability assays from treatments with cytochalasin B (CytB), colcemid (Col) or docetaxel (Doc) alone and in combination with 6 μM Tc52. Tc52 itself reduced viability to 77.1 % +/− 1.78 SEM. Tc52 together with CytB displays additive toxicity to CytB alone (parallel curves), whereas Tc52 acts protective in combination with high doses of Col or Doc, as evident from the Combination Index calculated by the algorithm of Chou and Talalay [38] (last panel, Col +/− 0.023 SEM, Doc +/− 0.032 SEM, compared to hypothetical value 1). **b**: Cell-cycle analysis of single and combinatory treatments after 24 h application of 4 μM cytochalasin B (CytB), 27 nM colcemid (Col) and 50 nM docetaxel (Doc) alone or in combination with 6 μM Tc52. Single treatments were compared to control or to the respective combination. Significance was calculated by two-way ANOVA with Dunnett's Multiple Comparison Test *p* < 0.05 = *, *p* < 0.01 = **, *p* < 0.001 = *** for comparison of treatments to control, and with two-tailed *T*-test between single and double treatments. Tc52 has no impact on cell-cycle profile, Col increases number of cells in G2/M (1.5-fold), accompanied by a drop in G1 (1.5-fold). This is more pronounced with CytB (1.8-fold and 28.8-fold, respectively) or Doc (2.3-fold and 23.3-fold, respectively). Cells in S-phase are significantly reduced, 6-fold with CytB and 3.6-fold with Doc. SubG1 is increased for Col (12.4-fold), CytB (6.8-fold) and Doc (17-fold). Tc52 addition does not change cell-cycle profile of Col treatment, but reduces number of cells with >4 N content in CytB and Doc treated samples 1.8-fold and 1.3-fold compared to single treatments, respectively. G2/M content and SubG1 are increased only compared to CytB treatment 1.4-fold and 1.3-fold, respectively. Filled bars: G1; Open bars: S; Hatched bars: G2/M; Pointed bars >4 N; Vertical line bars: subG1. **c**: Cells were treated for 24 h with toxins as in **3b**, fixed with formaldehyde and analyzed by DAPI staining for the presence of mitotic figures. 100 cells/experiment were evaluated (in docetaxel-treated samples only 50 cells/experiment) in independent triplicates. The percentage of mitotic cells (Mitotic Index, MI) was calculated and values compared to control (*). In addition, double treated samples were compared to single treatments (+). Significance was calculated by two-tailed *T*-test, *p* < 0.01 = **/++, *p* < 0.001 = ***/+++. CytB reduced MI 3.6-fold, Col and Doc enhanced MI 2.1-fold and 11.4-fold, respectively. Tc52 severely reduces MI in all cases. **d**: Immunofluorescence analysis of actin and tubulin in drug-treated HeLa cells. Cells were treated and fixed as described in **3c**. Tubulin was detected by indirect immunofluorescence whereas f-actin was directly detected by Atto488-coupled phalloidin. Control cells (Ctr) show normal mitosis (filled arrow) and proper actin and tubulin network. Tc52-treated samples display changes in cell morphology such as tubulin bundles at the cell periphery (open arrow) and alterations of f-actin pattern. Cytochalasin B (CytB) changes actin distribution, inducing either aggregation or dim f-actin staining as well as binuclear cells (asterisk). Tubulin is unaffected. Double treated samples (Tc52/CytB) show a combination of changes in f-actin as well as tubulin networks (open arrow), but lack bi-nuclear cells. Colcemid-treated cells (Col) display improper mitotic spindle formation and cells blocked in metaphase (filled arrow). Double treated samples (Tc52/Col) are free of mitotic figures and display altered tubulin network (open arrow). Docetaxel (Doc) induces high levels of mitotic cells (filled arrow) and lobed nuclei, accompanied by strong staining of short tubulin fibers and mitotic spindles. As cells are blocked in mitosis, it was not possible to evaluated changes in the actin network. Double treated samples (Tc52/Doc) completely lack mitoses, and arrangement of tubulin fibers is improved and cells display proper actin network

We performed flow-cytometry analyses of HeLa cells after 30 h and 48 h of continuous treatment with different Tc52 concentrations to monitor for potential interference with cell-cycle progression (Fig. 2, see Additional file 4: Figure S4 for representative histograms). We noticed a profound toxic effect (subG1-fraction, up to 30-fold induction) with increased concentration and incubation time, accompanied by loss of G1-cells (2-fold). At 2 μM and higher, G2/M content increased significantly (1.5-fold), but dropped below control values at 10 μM. Tc53 had no impact on cell-cycle profile. As Tc52-dependent G2/M-accumulation may have been the result from interference in mitotic progression, we combined Tc52 treatment with toxins blocking important steps in mitosis and cytokinesis, i.e., actin-targeting cytochalasin B (CytB) or tubulin-targeting colcemid (Col) and docetaxel (Doc). To study potential interactions between Tc52 and the toxins, viability assays in HeLa were performed using single agents or combinations with 6 μM Tc52 (EC_50_) for 24 h to reduce Tc52-dependent cell death (Fig. 3). Under these conditions, Tc52 induced a 23 % loss in viability. To analyze the interaction mode of the combined compounds (antagonistic/additive/synergistic), we employed the algorithm of Chou and Talalay [38]. As evident from Fig. 3a, cytotoxicity of CytB and Tc52 was independent, leading to additive loss of viability. Tc52 blocked toxicity at high concentrations of tubulin-targeting Col and Doc (antagonistic), suggesting that Tc52 may target the very same structure. We performed cell-cycle analysis in cells exposed to toxins alone or in combination, i.e., 6 μM Tc52, 4 μM CytB, 27 nM Col and 50 nM Doc, respectively (Fig. 3b, see Additional file 5: Figure S5 for representative histograms). Tc52 treatment for 24 h did not change cell-cycle profile, whereas Col-treated cells mildly accumulated in G2/M (1.5-fold), which was more pronounced by CytB (1.8-fold) and even further by docetaxel with more than 60 % in G2/M (2.9-fold). An increase of cells with a DNA content >4 N was detected in CytB-treated cultures (5.3-fold), most likely due to repressed cytokinesis, which leads to formation of binuclear cells. Doc also induced this fraction significantly (2-fold). All single treatments with CytB (6.7-fold), Col (12.3-fold) or Doc (16.9-fold) induced a subG1 peak, an indicator of cell death, and a reduction in G1 cell count (28.8-fold, 1.5-fold, and 23.2-fold, respectively) Tc52 addition reduced the percentage of cells with >4 N in Tc52-docetaxel samples compared to Doc alone (1.6-fold of control). The increase of cells with >4 N induced by CytB was severely decreased by 50 % with a concomitant increase in G2/M- and subG1-fractions (2.5-fold and 9.2-fold compared to control, respectively). To discriminate between mitosis and G2-phase, we treated cells as above and determined the mitotic index (MI) (Fig. 3c). As expected, Col and Doc caused an increase in MI by twofold and more than 11-fold, respectively. In contrast, CytB reduced MI more than threefold. Tc52 treatment alone induced a complete absence of mitotic figures. Combination of Tc52 with the toxins severely reduced the MI below normal control values, which indicates that Tc52 acts in advance of Col or Doc. To monitor the impact of Tc52 on cytoskeletal organization, we performed immunofluorescence co-staining for tubulin and actin (Fig. 3d). As expected, CytB induced binuclear cells (asterisk) with normal tubulin network, and actin was partially aggregated as detected by phalloidin-staining. Col blocked metaphases (closed arrow) with impaired mitotic spindles and normal actin network. Doc induced multipolar spindles in cells arrested in mitosis (closed arrow), and lobed nuclei in cells escaping it. Tubulin was strongly stained and arranged as straight fibers. Tc52-treated cells displayed slightly diminished phalloidin-staining with granules, and tubulin bundles in cellular protrusions (open arrows). Co-treatments showed deranged actin and tubulin networks. Induction of binuclear cells by CytB, metaphase arrest by Col, or fixed mitosis/lobed nuclei by Doc were no longer detectable when Tc52 was co-applied as expected. In summary, this indicates that Tc52 interferes with a step before progression into mitosis, i.e., during G2-phase.

### Tc52 induces apoptosis in cancer cells and senescence in fibroblasts

**Fig. 4:**
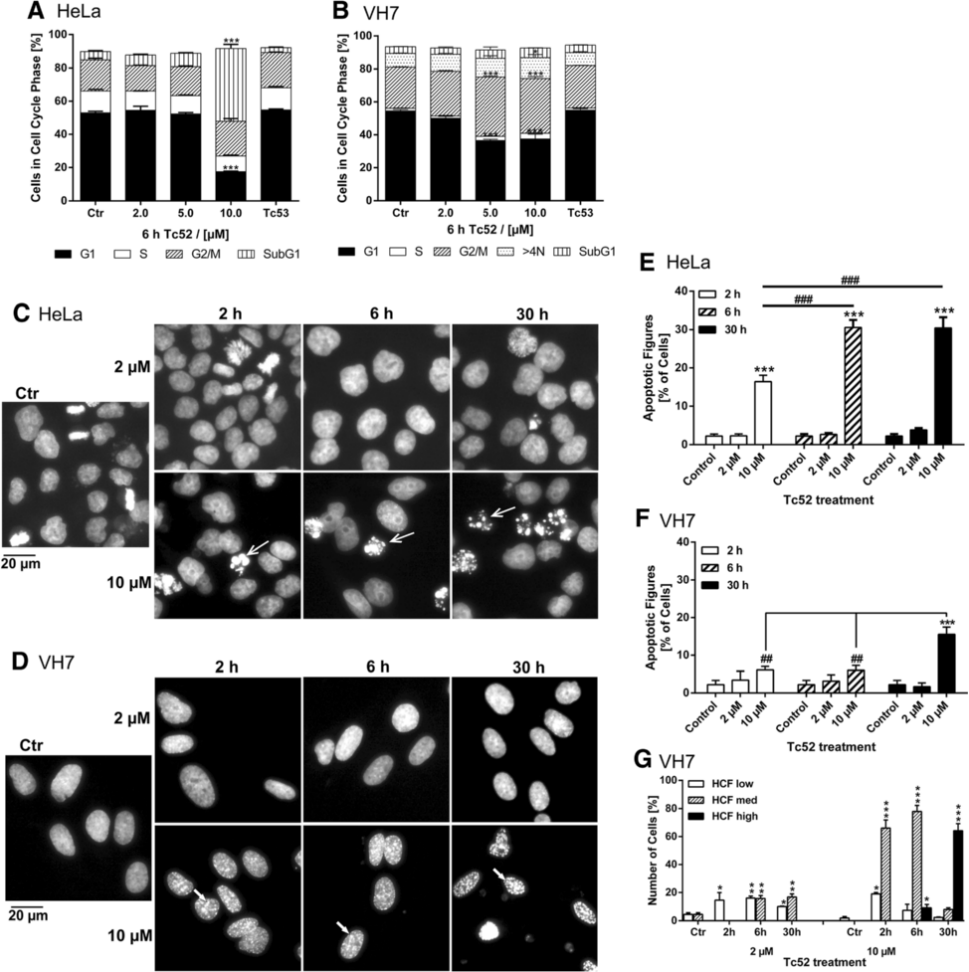
Tc52 induces apoptosis in HeLa but not in VH7 fibroblasts. HeLa and VH7 cells were exposed to three different Tc52 concentrations (2 μM, 5 μM, 10 μM) and 10 μM Tc53 for indicated time points, followed by incubation in toxin-free medium. Treatments were compared to controls and significance was calculated by two-way ANOVA with Dunnett's Multiple Comparison Test, *p* < 0.05 = *, *p* < 0.01 = **; *p* < 0.001 = ***. Significance between different time-points was calculated by one-way ANOVA with Tukey-Kramer Multiple Comparisons Test, *p* < 0.01 = ##; *p* < 0.001 = ###. **a**: Cell-cycle distribution of HeLa cells after 6 h Tc52 treatment and 24 h release reveals major increase in the subG1 fraction at 10 μM Tc52 (8.7-fold, accompanied by a loss of cells in G1 (3-fold). Tc53 has no effect. **b**: Cell-cycle distribution of VH7 after 6 h Tc52 treatment and 24 h release reveals accumulation in G2/M and increased >4 N-fraction accompanied by loss from G1 without increase in subG1 at 5 μM (1.5-fold G2/M, 1.4-fold >4 N, 1.5-fold G1) and 10 μM (1.3-fold G2/M, 1.5-fold >4 N, 1.3-fold G1) Tc52. Tc53 has no effect. **c**: Representative pictures of HeLa cells displaying dose- and time-dependent appearance of apoptotic figures (open arrows). Cells were treated with 2 μM or 10 μM Tc52 for 2 h, 6 h, or 30 h, washed, fixed with formaldehyde and nuclei were stained with DAPI. **d**: Representative pictures of normal VH7 fibroblasts displaying a dose- and time-dependent alteration of the nuclear structure, forming DAPI-rich foci (filled arrows). Cells were treated with 2 μM or 10 μM Tc52 for 2 h, 6 h, or 30 h, washed, fixed with formaldehyde and nuclei were stained with DAPI. **e**: Statistical evaluation of number apoptotic figures in HeLa cells from three independent experiments counting at least 100 cells each. Significant apoptosis induction compared to control (2.3 %) is detected in 10 μM Tc52 samples treated for 2 h (16.4 %) and rate increases with exposure to 6 h (30.6 % at 6 h and 30.5 % at 30 h). Significance of increase compared to control was analyzed by two-way ANOVA and Dunnett’s Multiple Comparison test (*), and differences between 10 μM treatments were calculated by one-way ANOVA and Tukey’s Multiple Comparison Test (#). **f**: Statistical evaluation of apoptotic figures in VH7 cells from three independent experiments counting at least 100 cells each. Significant increase in apoptotic cells compared to controls (2.2 %) is only detected in cultures exposed to 10 μM Tc52 for the complete 30 h (15.5 %). Significance of increase compared to control was analyzed by two-way ANOVA and Dunnett’s Multiple Comparison test (*), and differences between 10 μM treatment was calculated by one-way ANOVA and Tukey’s Multiple Comparison Test (#). **g**: Statistical evaluation of VH7 cells nuclei containing DAPI-rich foci. Foci were graded regarding their intensity over the nuclear DAPI background from three independent experiments counting at least 100 cells each. There is a clear dose- and time-dependent increase in the number of cells displaying DAPI-bright foci with a shift from low-grade to high-grade foci. Significance was calculated by two-way ANOVA and Dunnett’s Multiple Comparison Test (*)

**Fig. 5:**
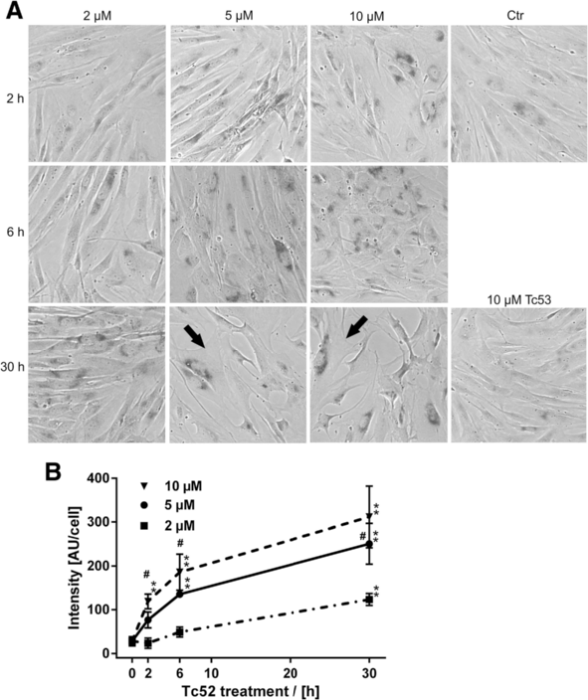
Tc52 induces senescence in VH7 fibroblasts. VH7 fibroblasts were treated as indicated in Fig. 4, including an additional 5 μM Tc52 concentration, for measuring SAβGal-activity. There is a clear increase in cells with active SAβGal as well as changes in cellular morphology, i.e., enlargement and flattening (panel **a**, black arrows). Lower panel **b** displays evaluation of SAβGal-activity. There is a significant dose- and time-dependent increase in SAβGal-activity per cell. Different doses at one time-point were compared and significance was calculated by one-way ANOVA with Bonferroni Multiple Comparison Test (#). Significant dose-dependency is detected at all time points except for 30 h, which shows difference between 2 μM and 5 μM or 10 μM, but not between 5 μM and 10 μM Tc52. Individual doses over time were compared to controls and significance was calculated by one-way ANOVA with Dunnett's Multiple Comparison Test, *p* < 0.01 = **

**Fig. 6:**
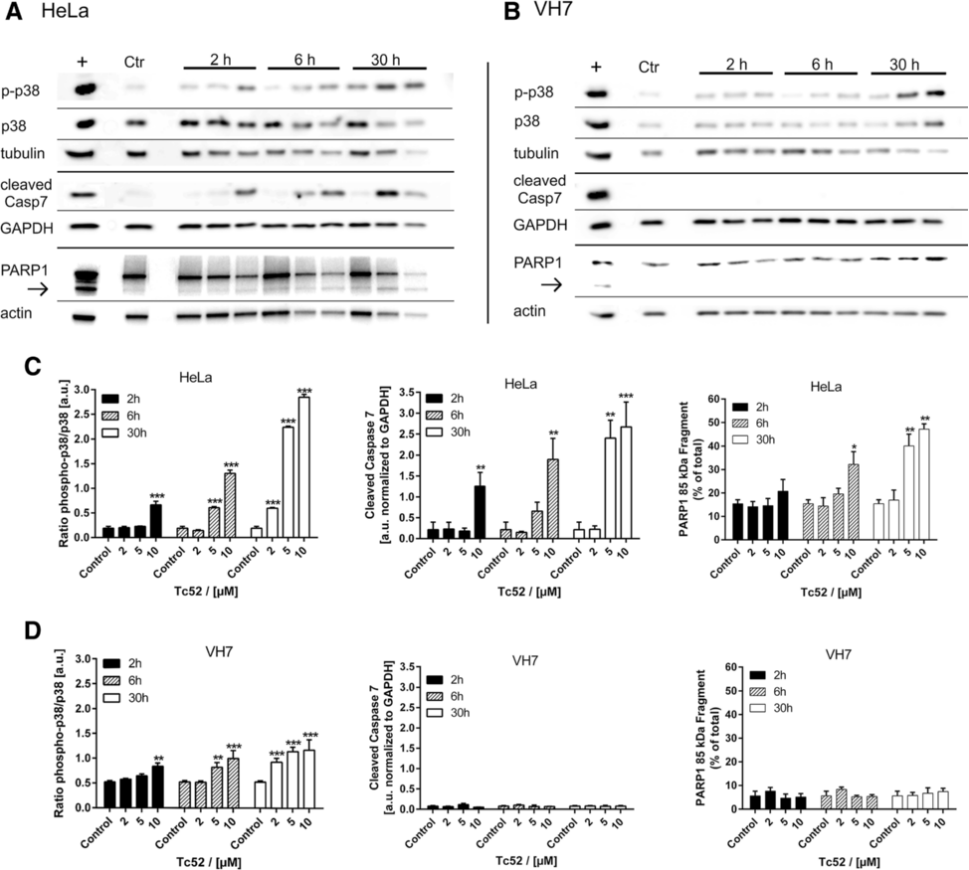
Activation of p38 stress-kinase, caspase7 and PARP1 cleavage by Tc52 in HeLa and VH7 fibroblasts. Treatment regimen was as described for Fig. 4. Ctr: solvent treated samples, +: positive control. Shown below each incubation time (2 h, 6 h, 30 h) are the different Tc52 concentrations used, i.e., 2 μM, 5 μM, and 10 μM. For statistical analysis, ratio of ECL signals of target protein compared to the respective loading control was calculated. Significant changes compared to solvent-control were analyzed by two-way ANOVA with Dunnett's Multiple Comparison Test, *p* < 0.05 = *; *p* < 0.01 = **; *p* < 0.001 = ***. **a**: Respective western blot panel for HeLa cells, displaying levels of phosphorylated p38 (p-p38) stress kinase, total p38 (p38), α-tubulin, cleaved caspase7, GAPDH, PARP1 and 85-kDa apoptotic fragment (arrow), and β-actin. **b**: Respective western blot panel for VH7 fibroblasts, displaying levels of phosphorylated p38 (p-p38) stress kinase, total p38 (p38), α-tubulin, cleaved caspase7, GAPDH, PARP1 and 85-kDa apoptotic fragment (arrow), and β-actin. **c**: Evaluation of stress-response in HeLa cells from at least three independent experiments. Panels display ratios of p-p38/p38, cleaved caspase7/GAPDH and PARP1 85 kDa/total, which all increase significantly in a time- and dose-dependent manner. **d**: Evaluation of stress-response in VH7 fibroblasts from at least three independent experiments. Panels display ratios of p-p38/p38, cleaved caspase7/GAPDH and PARP1 85 kDa/total. Only p38-dependent stress response increases significantly in a time- and dose-dependent manner without any signs of apoptotic alterations (cleavage of caspase7 or PARP1)

**Fig. 7:**
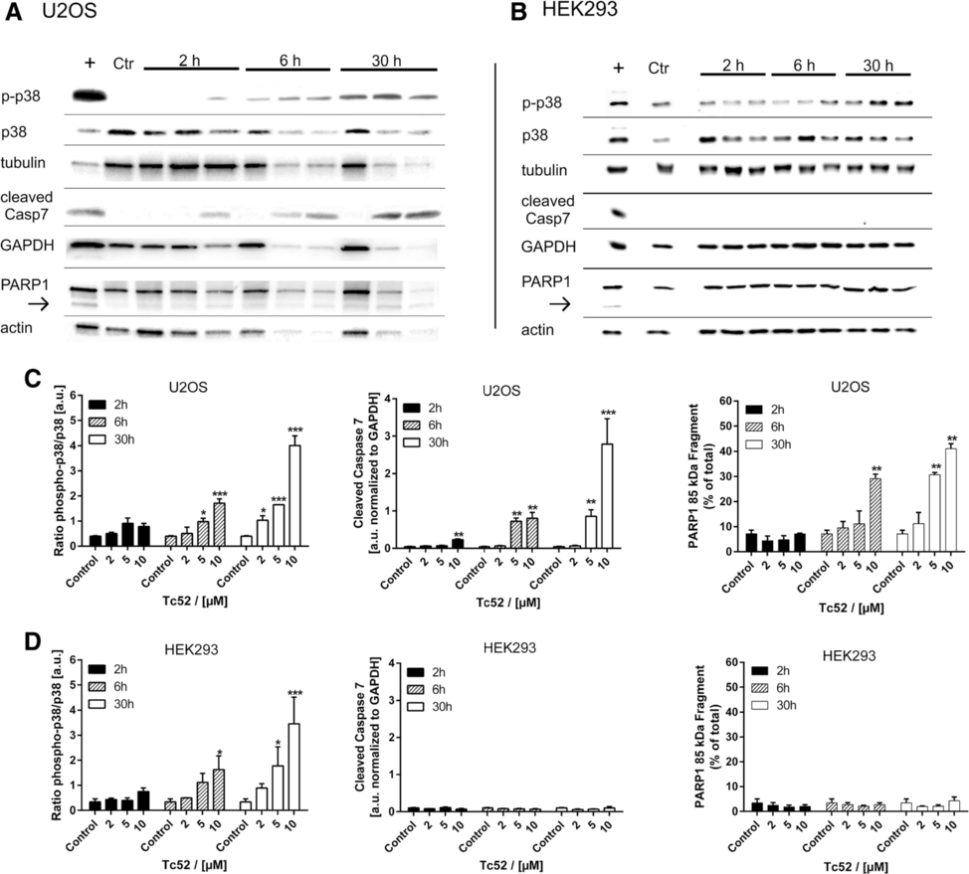
Activation of p38 stress-kinase, caspase7 and PARP1 cleavage by Tc52 in U2OS and HEK293. Treatment regimen was as described for Fig. 4. Ctr: solvent treated samples, +: positive control. Shown below each incubation time (2 h, 6 h, 30 h) are the different Tc52 concentrations used, i.e., 2 μM, 5 μM, and 10 μM. For statistical analysis, ratio of ECL signals of target protein compared to the respective loading control was calculated. Significant changes compared to solvent-control were analyzed by two-way ANOVA with Dunnett's Multiple Comparison Test, *p* < 0.05 = *; *p* < 0.01 = **; *p* < 0.001 = ***. **a**: Respective western blot panel for U2OS cells, displaying levels of phosphorylated p38 (p-p38) stress kinase, total p38 (p38), α-tubulin, cleaved caspase7, GAPDH, PARP1 and 85-kDa apoptotic fragment (arrow), and β-actin. **b**: Respective western blot panel for HEK293 cells, displaying levels of phosphorylated p38 (p-p38) stress kinase, total p38 (p38), α-tubulin, cleaved caspase7, GAPDH, PARP1 and 85-kDa apoptotic fragment (*arrow*), and β-actin. **c**: Evaluation of stress-response in U2OS cells from at least three independent experiments. Panels display ratios of p-p38/p38, cleaved caspase7/GAPDH and PARP1 85 kDa/total, which all increase significantly in a time- and dose-dependent manner. **d**: Evaluation of stress-response in HEK293 from at least three independent experiments. Panels display ratios of p-p38/p38, cleaved caspase7/GAPDH and PARP1 85 kDa/total. Only p38-dependent stress response increases significantly in a time- and dose-dependent manner without any signs of apoptotic alterations (cleavage of caspase7 or PARP1)

In a next step, we investigated whether Tc52 pulse-treatment might be sufficient to induce these effects (Fig. 4). We incubated HeLa and VH7 for 6 h with increasing Tc52 concentrations and analyzed after 30 h the cell-cycle distribution by flow-cytometry. Whereas HeLa displayed major cytotoxicity at 10 μM with a loss of G1-cells (3-fold), fibroblasts showed no cell death as indicated by an unchanged subG1-fraction. Instead, cells in G1 gradually decreased (1.4-fold) concomitant with an accumulation in G2/M (1.5-fold) (Fig. 4a/b, see Additional file 6: Figure S6 for representative histograms), suggesting a differential response to Tc52 in tumorigenic HeLa and untransformed VH7 fibroblasts. To analyze this in more detail, HeLa and VH7 were treated for 2 h, 6 h, or 30 h with 2 μM or 10 μM Tc52 and nuclei were monitored for apoptosis or other alterations after a total of 30 h (Fig. 4c-f). HeLa displayed after the 2 h exposure to 10 μM Tc52 a sevenfold increase in apoptotic figures (Fig. 4c/e), which doubled with prolonged treatment, but no additional changes in nuclear morphology. VH7 displayed only at 30 h of 10 μM Tc52 a minor increase in apoptotic cells (Fig. 4d/f). In contrast, chromatin in VH7 nuclei structurally changed and DAPI-dense regions appeared, dependent on Tc52 concentration and exposure time (Fig. 4g). These foci very much resembled senescence associated heterochromatic foci (SAHF) [39, 40], which are indicators of cellular senescence. To further investigate on this, we analyzed senescence-associated beta-galactosidase (SAβGal)-activity, an enzymatic hallmark of senescence [40, 41]. Using the same pulse-treatment setup as before, we observed a time- and dose-dependent cell-enlargement, accompanied by increasing SAβGal-activity (Fig. 5a). Tc53-treated cells were undistinguishable from controls. There was a clear gain in positive staining with time and concentration of Tc52 (Fig. 5b). These results provide solid evidence that Tc52 induces senescence in fibroblasts. To support our observations about cell-specific toxicity of Tc52 by an alternative approach, we employed a recently established method to analyze cytotoxic-potential of compounds by measuring changes in free cytosolic Ca^2+^-levels by Fluo4-NW-dye [42]. We exposed HeLa and VH7 to 2 μM or 10 μM Tc52 or 10 μM Tc53, and monitored for increased free cytosolic Ca^2+^ (Additional file 7: Figure S7, for methods see Additional file 8). In line with the cell-cycle and DAPI analysis, HeLa displayed a rapid increase in Ca^2+^-dependent fluorescence at both Tc52 concentrations (Δ4-RFU and Δ5.5-RFU after 5 s/ Δ6.5-RFU and Δ5-RFU after 10 s, respectively). In contrast, free Ca^2+^ in VH7 increased only marginally and very transient over control at 10 μM (Δ1.1-RFU after 5 s, Δ0-RFU after 10 s). 10 μM Tc53 was indistinguishable from controls. To strengthen our observation of cell-line specific response in toxicity, we treated cultures with Tc52 or Tc53 for 30 h and analyzed phosphorylation of the stress-associated p38 MAP-kinase (p-p38) and the appearance of cleaved caspase7, a sign for ongoing apoptosis. Both HeLa and VH7 showed an elevation in p-p38, but only HeLa displayed pronounced induction of active caspase7 (Fig. 6a-d), whereas Tc53 induced neither p-p38 nor caspase7-cleavage (Additional file 9: Figure S8). To validate these data, we monitored the cleavage of poly(ADP-ribose) polymerase1 (PARP1), a well-established event in early execution phase [43, 44]. Apoptotic PARP1 85 kDa-fragment was significantly induced in HeLa, but not in VH7 fibroblasts (Fig. 6). Identical response-patterns were observed in U2OS cancer cells and low-passage HEK293 (Fig. 7), with the latter being immortal but non-tumorigenic [45]. Viability loss induced by Tc52 was similar compared to HeLa or VH7, with EC_50_ values of 2 μM (U2OS) and 4 μM (HEK293) (Additional file 10: Figure S9). In both cell lines p-p38 increased in a dose- and time-dependent manner, but only U2OS cells displayed activation of caspase7 and PARP1-cleavage (Fig. 7). In summary, Tc52 induced a p-p38 related stress signaling in all cell lines, but only cancer cells responded with apoptosis.

## Discussion

Efficacy of cancer treatment relies on higher sensitivity of tumor cells towards chemotherapeutic agents compared to surrounding tissue. The search for metal-based chemotherapeutics other than cisplatin-derivatives led to the discovery of titanium as potential replacement, with titanocene-dichloride and budotitane reaching clinical trials. Unfortunately, both compounds lacked anti-tumorigenic potential due to rapid hydrolysis in aqueous solutions [28]. Titanium(IV)salans are much more stable [29] and have been shown to display promising activity against cancer cells in vitro as well as in mouse models, including some selectivity for tumor cells [30]. But the underlying mechanism of cytotoxicity and the cellular targets have not been defined yet. We investigated this for the toxic titanium(IV)salan Tc52 and the non-toxic Tc53, which display a high degree of structural similarity with only small variations, in different human cell lines. Viability was similarly affected by Tc52 in all lines analyzed, with EC_50_ values between 2 μM and 6 μM Tc52 (Additional file 2: Figure S2 and Additional file 10: Figure S9), whereas Tc53 had no impact. This discrepancy in toxicity cannot be attributed to differential uptake, as similar timing in cellular accumulation has been reported for related titanium(IV)complexes [46]. Importantly, viability assays do not discriminate between cytotoxic and cytostatic effects, as in both cases cell numbers are reduced compared to controls. Deduced from titanocene-dichloride [33], we expected genomic DNA to be the target of Tc52. We monitored for DNA strand-break induction with a variety of different assays, using the very sensitive FADU and reverseFADU method able to detect strand-breaks induced by 0.13 Gy of X-ray [36] as well as DNA crosslinks [37], respectively, and checked for the appearance of the two different damage markers PAR and γH2AX. Whereas synthesis of PAR is an early marker for DNA strand-breaks, detectable within seconds to minutes [47], γH2AX foci appear later and correlate finally with the amount of double-strand breaks [48]. We failed to observe any DNA damage induction (Fig. 1) after Tc52 application in contrast to H_2_O_2_ treatment (Additional file 3: Figure S3). These data exclude that nuclear DNA is targeted by Tc52. Analyzing the cell-cycle profile of HeLa after Tc52 treatment revealed major cell death induction concomitant with a transient block in G2/M-phase (Fig. 2). A reasonable explanation could be that Tc52 interferes with a step during mitosis or cytokinesis, similar to biological toxins such as cytochalasin B from fungi, colchicine from autumn crocus or taxanes from yew. CytB induces binuclear cells as it interferes with formation of the contractile actin-ring in cytokinesis. In contrast, the colchicine-derivate colcemid or the taxane docetaxel target tubulin, leading to metaphase arrest, but whereas Col prevents tubulin polymerization, Doc blocks disassembly of tubulin. As a consequence of taxane treatment, multipolar spindles are induced, and cells escaping mitotic block show characteristic lobed or fragmented nuclei [49]. Tc52 was applied in combination with all three compounds. Tc52 toxicity was additive to CytB in viability assays, but protected from cytotoxicity of higher doses of Col and Doc (Fig. 3a). Whereas addition of Tc52 mildly changed cell-cycle profile of Col and Doc treated samples, the percent of cells containing >4 N DNA content, i.e., binucleated cells, was drastically reduced in CytB/Tc52-treated cells (Fig. 3b). DAPI-staining of nuclei revealed that addition of Tc52 led to a severe drop in mitotic index (Fig. 3c). In concert with the cell-cycle analysis, these data indicate that Tc52 is not affecting mitosis, but targets an unknown step in G2, preventing progression into M-phase. We hypothesize that steps before or at G2-M transition as centrosome-separation, kinase-signaling or the cytoskeleton might be affected. Interestingly, it has been reported that tubulin targeting colcemid has two different modes of action: At low doses it inhibits proper plus-end dynamics and impacts on cell migration, whereas at high doses it seems to induce cell death by microtubule fragmentation during mitosis [50]. Indeed, visualization of tubulin and F-actin indicated that Tc52 induced small alterations in the tubulin network, displaying increased bundling at the cellular periphery also in combination with other toxins (Fig. 3d). Whether Tc52 also impacts on migration needs to be determined in future research. But probably Tc52 acts independent of this as treated cells are blocked in a step before condensation of chromosomes takes place and mitotic microtubules are formed. Actin was mildly affected by Tc52, which could be due to modified tubulin functionality. F-actin staining was severely affected by CytB as expected, reshuffling actin into bright foci without network-formation. Col and Doc affected only the tubulin network. Of note, combined Doc/Tc52 treatment "rescued" actin and tubulin network compared to Doc-only treated samples, which supports the idea of acting in advance of Doc and thus mitosis. In summary, the protection from Col- and Doc-dependent cytotoxicity, together with the cell-cycle analysis and data from immunofluorescence suggests that Tc52 acts on the same pathway as Col or Doc, but independent of actin-targeting CytB. Monitoring cell-cycle distribution after Tc52 pulse-treatment of HeLa and VH7 revealed massive apoptosis-induction in tumorigenic HeLa, whereas fibroblasts were blocked in G2/M without signs of cell death (Fig. 4a/b). Appearance of apoptotic figures in HeLa confirmed the time- and dose-dependent increase in toxicity by Tc52 (Fig. 4c/e), which was absent in VH7 as expected from cell-cycle data (Fig. 4d/f). Instead, VH7 nuclei displayed changes in chromatin structure, i.e., an increase of DAPI-dense foci resembling SAHF [39, 40] (Fig. 4d/g), together with characteristic morphological changes such as enlargement and flattening (Fig. 5). Testing for SAβGal activity confirmed a time- and concentration-dependent induction of senescence (Fig. 5). To support our findings, we analyzed the rapid release of free Ca^2+^ in the cytosol in HeLa and VH7 after Tc52 exposure. This method was developed to quickly determine the cytotoxic potential of a substance [42]. Both low and high concentrations induced in HeLa a strong increase in Ca^2+^-dependent fluorescence, which was absent in fibroblasts (Additional file 7: Figure S7), supporting the cell-specific difference in Tc52 response. Activation of the general stress-kinase p38 by phosphorylation at T180/Y182 is central to many cell fate pathways such as cell death, senescence, differentiation and tumorigenesis (reviewed in [51]). In order to analyze whether Tc52 and Tc53 application leads to activation of p38, we probed for total and phosphorylated p38 in four cell lines. Indeed, Tc52 led to increased p-p38 in a time and dose-dependent manner in all of them (Figs. 6, 7). In contrast, non-cytotoxic Tc53 did not show enhanced p-p38 levels (Additional file 8: Figure S8). To confirm the observed differential impact of Tc52 on cell death, we probed for apoptosis-dependent cleavage of caspase7 [52]. Indeed, only HeLa and U2OS displayed dose- and time-dependent activation of caspase7 (Figs. 6, 7), and Tc53 was without effect (Additional file 9: Figure S8). Supporting our data, apoptotic PARP1-cleavage was evident only in HeLa and U2OS cultures treated with Tc52, but not in VH7 and HEK293 (Figs. 6, 7).

In summary, the titanium(IV)salan Tc52 is effective with an EC_50_ value in the low-micromolar range. Tc52 interferes with cell-cycle progression in G2 and deduced from our data, tubulin-related signaling is targeted, as Tc52 treatment induces alterations in the tubulin network and partially rescues from cytotoxicity of tubulin-interacting toxins that induce mitotic arrest. Alternatively, Tc52 may interfere with centrosomal regulation and prevent in this way entry into mitosis, but this would not explain the differential response towards combination with actin- or tubulin-targeting toxins. The actual target of Tc52 has to be elucidated in future research. Nevertheless, as a consequence tumor cells respond with apoptotic death, whereas normal fibroblasts show induction of senescence. We hypothesize that the constant block in G2 imposed by Tc52 cannot be handled properly by tumor cells, leading to induction of apoptosis.

## Conclusions

We provided several layers of evidence for the differential cytotoxic potential of the titanium(IV)salan Tc52 by a pathway that is not affecting genomic integrity. As proof-of-principle, we compared two well-established cancer cell lines, HeLa and U2OS, with VH7 fibroblasts and low-passage HEK293. Importantly, Tc52 does not target DNA or block progression through mitosis, which maintains genomic information and stability. In this way, there is no risk of inducing mutations in normal tissue, which may lead to the outgrowth of secondary tumors after cancer therapy [53]. P38 stress kinase is activated in all cells, but only tumor cell lines responded with cell death as evidenced by caspase7 activation and PARP1-cleavage. Supporting this, HeLa cells treated with Tc52 displayed an increase in subG1-fraction, apoptotic figures and cytosolic Ca^2+^, but not human fibroblasts, which showed induction of senescence. Further exploration of the potential anti-tumorigenic effect of this new titanium(IV)salan could be of great clinical benefit. If chemotherapy would actually discriminate between tumor and normal cells, fighting cancer could be far more effective.

## Methods

### Cell culture and toxins

Cells were cultured in DMEM-GlutaMax (Life-Technologies)/10 % FCS/1 % PenStrep (Life-Technologies). VH7 normal fibroblasts were used from passage 20–30, HEK293 from passage 24–34, HeLa and U2OS from routine culture. Authentication of cell lines was performed by short tandem repeat (STR) DNA-typing. Toxins were applied in medium at the concentrations and for the time periods specified with appropriate solvent controls in parallel. DMSO concentrations in medium were either 0.2 % (titanium(IV)salans alone) or 0.3 % in combination treatments. Titanium(IV)salans (Chemistry-Department University of Konstanz), cytochalasinB (Sigma) and docetaxel (LC-Laboratories) were solubilized in DMSO, colcemid (KaryoMax, Life-Technologies) and H_2_O_2_ (Sigma) diluted in medium.

### Viability assay

Cells were seeded into 96well-plates (clear bottom/white walls, Corning) and grown for 24 h. Medium was exchanged against medium containing toxins and incubated for 24 h or 48 h. Supernatant was replaced with fresh medium containing 9 μg/ml resazurin (Sigma) and incubated until medium in control wells turned purple. Fluorescence (excitation/emission 560 nm/590 nm) was measured using LS55-spectrometer (Perkin-Elmer). Each experiment was performed in technical duplicates or triplicates.

### Immunofluorescence

Cells were seeded 24 h in advance onto glass cover-slips. Medium was supplemented with the respective toxin and incubated for the times specified. Cells were fixed with 3.7 % formaldehyde/PBS for 20 min at room temperature (RT), washed 1x with 0.1 M glycine/PBS for 3 min at RT and permeabilized for 5 min at RT in 0.4 % TritonX100/TBS except for detection of PAR. For PAR, cells were fixed by incubation for 7 min in −20 °C methanol at 4 °C. Subsequent procedures were identical. After three washes with TBS, cells were blocked in TBS/0.3 % Tween 20/1 % BSA for 30 min at 37 °C and incubated for 1 h at 37 °C with 1.antibody diluted in blocking-solution, followed by three washes for 10 min at RT in TBS/0.3 % Tween 20. 2.antibody incubation was performed for 1 h at 37 °C in blocking-solution, followed by three washes as above. Nuclei were stained with 20 ng/ml DAPI-solution, air-dried and mounted with AquaPolymount (PolySciences). Epifluorescence pictures were taken using a Nikon-EclipseTS100 microscope and NIS-ElementsD3.2 software. Subsequent analysis was performed using ImageJv1.47n and CS-Photoshop5.

Antibodies: 10H anti-PAR [54], anti-γH2AX (Millipore), anti-αTubulin (Sigma), goat-anti-mouse Alexa594 (Molecular Probes, Invitrogen). Phalloidin-Atto488 anti-F-actin (Sigma) was solubilized in DMSO to 40 μM and further diluted to 400 nM.

### Automated FADU and reverseFADU

Methods have been described in detail in [36, 37]. Briefly, cells were seeded 24 h before toxin treatment. After incubation with respective concentrations of mitomycinC, 10 μM Tc52 or Tc53 for the indicated times, cells were harvested and analyzed for strand-breaks by FADU or in parallel for crosslinks by reverseFADU. For reverseFADU, cells were additionally irradiated with 25 Gy X-rays before FADU procedure. Liquid handling was performed on a Genesis RSP100 robot (Tecan). Cells were lysed and unwinding of DNA was induced by alkaline conditions. After neutralization, the relative amount of double-stranded DNA in each sample was measured by SYBR-Green fluorescence (excitation/emission 492 nm/520 nm) in FLx800-fluorescence microplate-reader (Bio-TEK Company).

### Cell-cycle analysis

Cells were seeded on 10 cm-dishes and incubated for 24 h. Medium was replaced with medium containing respective toxins and incubated for the time periods indicated. In samples exposed for 6 h, medium was replaced with fresh standard medium and further incubated for 24 h. Floating cells were collected, combined with adherent cells harvested by trypsin, and pelleted by centrifugation. Cells were washed twice with ice-cold PBS, fixed by resuspending in ice-cold 70 % ethanol to a concentration of 1x10^6^ cells/ml and stored at −20 °C. For analysis, fixed cells were pelleted by centrifugation and washed once with PBS. Pellets were suspended in PBS containing 100 μg/ml RNaseA and 25 μg/ml propidium iodide to 1x10^6^ cells/ml. Cell-cycle distribution was monitored using FACSCantoII (BD-Biosciences) and about 10000 events were counted in each single run. Subsequent analysis was performed using Flowing-Software2.1.

### SAβGal activity assay

The SAβGal-kit from Cell-Signaling was used following manufacturer's instructions. VH7 were seeded into 6well-plates and incubated for 24 h. Afterwards, medium was exchanged against medium containing Tc52 at concentrations of 2 μM/5 μM/10 μM, 10 μM Tc53 or solvent. Cells were incubated for 2 h/6 h/30 h with Tc52 before exchange of medium against standard medium. After a total incubation of 30 h, cells were fixed and SAβGal-activity assay was performed. To analyze signal intensity, bright-field photomicrographs were taken with fixed exposure time using Nikon EclipseTS100 microscope and gray-values were measured with ImageJv1.47n-software. Nuclei were counted to normalize for cell number, and gray-values/cell were calculated.

### Western blotting

Cells were seeded on 10 cm-dishes and incubated for 24 h. Medium was replaced with medium containing respective toxins and incubated for the indicated times. After 2 h and 6 h medium was exchanged in the respective samples against fresh standard medium. After 30 h incubation of all samples, medium was removed and floating cells were harvested by centrifugation. Pelleted cells and cells on the dish were lysed in 95 °C Laemmli-buffer and combined. Equal volumes were run on SDS-gels and blotted onto nitrocellulose- (PARP1 detection) or PVDF-membrane. Membranes were blocked with TBS/0.1 % Tween20/3 % BSA/10 % non-fat dry-milk for 2 h at 37 °C and incubated overnight at 4 °C with 1.antibody diluted in TBS/0.1 % Tween20/3 % BSA. Membranes were washed thrice for 20 min at RT in TBS/0.1 % Tween20/3 % non-fat dry-milk and incubated for 1 h at RT with 2.antibody in TBS/0.1 % Tween20/3 % non-fat dry-milk. After three washes in TBS/0.1 % Tween20, ECL detection was performed (Pierce ECL-SuperSignal-Femto, ThermoFisher Scientific). Antibodies: rabbit-polyclonal anti-GAPDH (Ambion); mouse-monoclonal anti-actin (Millipore); rabbit-polyclonal anti-p38 kinase, rabbit-monoclonal anti-phospho-p38 kinase, rabbit-polyclonal anti-tubulin, mouse-monoclonal anti-cleaved caspase7 (Cell-Signaling); goat-anti-rabbit IgG-HRP, goat-anti-mouse IgG-HRP (Sigma), mouse-monoclonal anti-PARP1 C-2-10 (SantaCruz-Biotechnologies).

### Statistics

All experiments were performed at least three times independently. Statistics were calculated using GraphPad-Prism5 software and tests suggested by the program were used. Presented are means +/− SEM.

## Abbreviations

Col, colcemid; CytB, cytochalasin B; DAPI, 4',6-diamidino-2-phenylindole; Doc, docetaxel; FADU, Fluorometric Analysis of DNA Unwinding; MI, mitotic index; MMC, mitomycin C; PAR, poly(ADP-ribose); PARP1, poly(ADP-ribose)polymerase1; p-p38, phosphorylated p38 stress-kinase; RT, room temperature; SAHF, senescence-associated heterochromatic foci; SAβGal, senescence-associated beta-galactosidase

## Additional files

Additional file 1: Structure of Tc52 and Tc53. Top structure highlights the identical backbone of Tc52 and Tc53. R (circled in red) indicates the two different side-chains of Tc52 (methyl-group) and Tc53 (tert-butyl-group), respectively. Depicted below are the separate structures for Tc52 and Tc53. (PDF 1085 kb) Additional file 2: Cell viability assay with HeLa tumor cells (squares) and VH7 normal fibroblasts (circles). Cells were exposed to increasing concentrations of Tc52 (filled) or Tc53 (open) titanium(IV)salan compounds solubilized in DMSO and incubated for 48 h. Subsequently, medium was replaced by fresh medium containing 9 μg/ml resazurin and cells were further incubated. Viability is expressed as % of solvent control. Whereas Tc53 is non-toxic in the tested concentration range, Tc52 impairs viability in HeLa and VH7 cells with an EC20 of 3 μM and 1 μM, an EC50 of 6 μM and 3 μM, respectively, and an EC80 of about 10 μM for both. (PDF 1085 kb) Additional file 3: Detection of DNA strand break markers poly(ADP-ribosyl)ation and γH2AX in HeLa and VH7. **A**: PAR can be weakly detected in HeLa cells about 5 min after application of 500 μM H2O2 in cell culture medium, peaking at 10 min, and signals disappear completely after 60 min. Later time points are not depicted. Phosphorylated H2AX appears after 10 min incubation as a pan-nuclear signal, with pronounced characteristic foci formation after 6 h. **B**: PAR can be weakly detected in VH7 cells about 5 min after application of 500 μM H2O2 in cell culture medium, peaking at 30 min and signals disappear completely after 60 min. Later time points are not depicted. Phosphorylated H2AX appears after 10 min incubation as a pan-nuclear signal, with less well pronounced characteristic foci formation after 60 min. **C**: H2AX and-tubulin detection in a time-course from 0 min (Ctr) to 1440 min (24 h) after application of 500 μM H2O2 (H), 10 μM Tc52 (52) or 10 μM Tc53 (53) to HeLa cells. Signal-ratio evaluation of γH2AX/tubulin is presented in the lower panel. γH2AX formation in H2O2 treated samples is evident after 30 min and increases over time. Only after 24 h of Tc52 treatment, a mild increase in γH2AX is detectable, concurrent with the onset of cell death (Fig. 1a). (PDF 1085 kb) Additional file 4: Cell cycle distribution profile of HeLa cells after continuous Tc52 treatment. Representative histograms from data presented in Fig. 2. **A**: Cell-cycle distribution after 30 h of continuous treatment of cells with 1-10 μM Tc52 (concentration range showing an effect). Note the small (not significant) reduction in G1 with 2 μM Tc52 and the strong reduction with 5 μM and 10 μM Tc52 concomitant with a substantial increase in the subG1 fraction. The sample treated with 5 μM Tc52 displays in addition an increased number of cells in G2, whereas cells treated with 10 μM show a reduction in G2 phase. **B**: Cell-cycle distribution after 48 h of continuous treatment of cells with 1-10 μM Tc52 (concentration range showing an effect). Note the reduction in G1 with 1-2 μM Tc52 and the nearly complete loss in samples treated with 5 μM and 10 μM Tc52. SubG1 is increased in all samples and cells treated with 1 μM Tc52 display a significant increase in G2 phase. (PDF 1085 kb) Additional file 5: Cell cycle distribution profile of HeLa cells after treatment with M-phase targeting toxins in combination with Tc52. Representative flow-cytometry histograms from data presented in Fig. 3. For better visibility, only one M-phase targeting toxin with or without 6 μM Tc52 is displayed in each panel. There is no significant difference in cell-cycle profiles of Tc52 or control samples except a small increase G2-phase in Tc52 treated cells (upper left panel). CytB-treated samples show a near-complete loss of G1-peak and a reduction in G2, concomitant with an increase in subG1 and the appearance of a substantial cell-fraction with a >4 N DNA content with a strong peak at about 8 N (compare black graphs from upper right and upper left panel). Combination with Tc52 reduces this 8 N peak and increases the number of cells in G2 and slightly the number of cells in G1 (upper right panel). Col-treatment induces a reduction in G1-phase (compare the black graphs from lower left and upper left panel) concomitant with an increase in G2 and subG1 (lower left panel). Combination with Tc52 does not change the cell-cycle profile significantly. 50 nM Doc induces a complete loss of cells from G1, concomitant with an increase in subG1 fraction, number of cells in G2 as wells as cells with a > 4 N DNA content (compare black graphs from lower right panel with upper left panel). Addition of Tc52 decreased the number of cells with a > 4 N DNA content (lower right panel). (PDF 1085 kb) Additional file 6: Cell cycle distribution profile of cells after 6 h of treatment. Representative flow-cytometry histograms from data presented in Fig. 4. For better visibility, surface of the graph from Ctr sample (black line) is dotted. **A**: Cell-cycle distribution of HeLa cells after 6 h incubation with Titanium(IV)salan complexes Tc52 and Tc53 30 h after treatment start. Only 10 μM Tc52 shows significant impact on cell-cycle profile, i.e. severe reduction in G1 and increase of the subG1 fraction. **B**: Cell-cycle distribution of VH7 normal fibroblasts after 6 h incubation with Titanium(IV)salan complexes Tc52 and Tc53 30 h after treatment start. Samples exposed to 5 μM and 10 μM Tc52 display major reduction in number of cells in G1 concomitant with a mild increase in G2-phase. SubG1 fraction is not significantly elevated. (PDF 1086 kb) Additional file 7: Analysis of rapid free cytosolic Ca2 + -shifts in HeLa tumor cells and VH7 normal fibroblasts. Cells were processed as suggested by the manufacturer. Tc52 or Tc53 or solvent control (CTR) was administered to the cells and Ca2 + -dependent increase in fluorescence of Fluo4-NW-dye was measured. Significant increase in fluorescence is an indicator for cell death signaling. **A**: Increase in free cytosolic Ca2+ in HeLa cells. Both concentrations show significant and rapid increase in Ca2+ signal, reaching after 5 sec 4 and 5.5 RFU above CTR and after 10 sec 6.5 and 5 RFU above CTR, whereas control and Tc53 values stay low. **B**: No significant increase in free cytosolic Ca2+ in VH7 cells. Only the 10 μM Tc52 exposure samples display initially a mild increase (1 RFU above CTR), which is lost after 10 sec (0 RFU above CTR). As summary, rapid Ca2+ increase is indicative for cytotoxicity of Tc52 in HeLa cells, whereas its toxicity is negligible in VH7 normal fibroblasts, in line with other data. (PDF 1085 kb) Additional file 8: Additional Methods. (PDF 144 kb) Additional file 9: Analysis of p38 kinase and caspase activation by Tc53 in HeLa and VH7 cells. Cells were exposed to 10 μM Tc53 or solvent for 30 h and subsequently lysed in Laemmli buffer. Western blot analysis was performed as described for Figs. 6 and 7. There is no evidence for activation of p38 in. HeLa or VH7 cells (**A**) or cleavage of caspase7 (**B**). Significance was tested using two-tailed T-test. Panel (**C**) depicts the respective western blot for cleaved caspase7 (cleaved Casp7), GAPDH, total p38 stresskinase (p38), phosphorylated p38 (p-p38) and αtubulin (Tubulin). +: positive control, C: solvent control samples, T: 10 μM Tc53 treated samples. Numbers indicate respective independent experiments. (PDF 1085 kb) Additional file 10: Cell viability assay with U2OS tumor cells (squares) and low-passage HEK293 (circles). Cells were exposed to increasing concentrations of Tc52 (filled) or Tc53 (open) titanium(IV)salan compounds solubilized in DMSO and incubated for 48h. Subsequently, medium was replaced by fresh medium containing 9 μg/ml resazurin and cells were further incubated. Viability is expressed as % of solvent control. Whereas Tc53 is non-toxic in the tested concentration range, Tc52 impairs viability in U2OS and HEK293 cells with an EC20 of 1.5 μM and 2.5 μM, an EC50 of 2 μM and 4 μM, and an EC80 of about 4 μM and 20 μM, respectively. (PDF 1085 kb)
