# Supplementary figures and images for: Differential cytotoxicity induced by the Titanium(IV)Salan complex Tc52 in G2-phase independent of DNA damage
Source: BMC Cancer. 2016 Jul 13;16:469. doi: 10.1186/s12885-016-2538-0 (PMC4944496; doi:10.1186/s12885-016-2538-0)

## Additional Figure 1

### Structure of Tc52 and Tc53

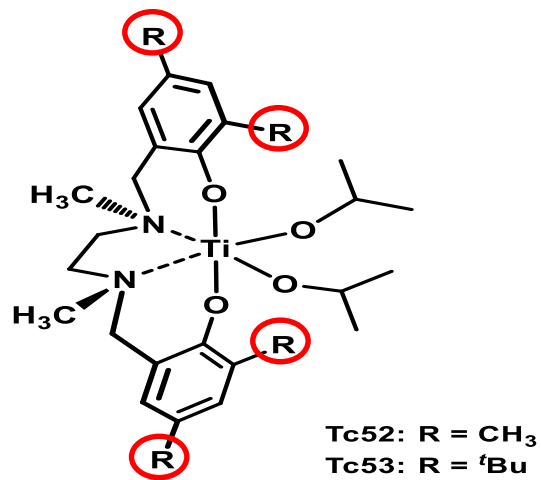

common structure

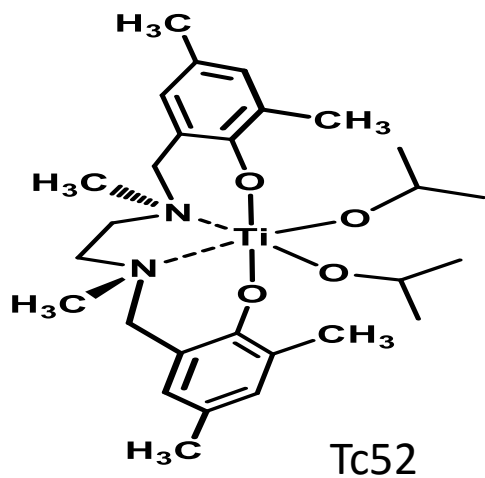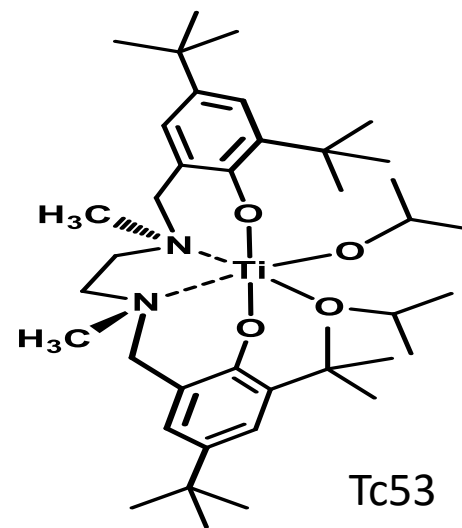

Supplement: Additional file 1: — Structure of Tc52 and Tc53. Top structure highlights the identical backbone of Tc52 and Tc53. R (circled in red) indicates the two different side-chains of Tc52 (methyl-group) and Tc53 (tert-butyl-group), respectively. Depicted below are the separate structures for Tc52 and Tc53. (PDF 1085 kb) [file 12885_2016_2538_MOESM1_ESM.pdf]

Additional Figure 2

Viability after 48 h Tc52 or Tc53 application

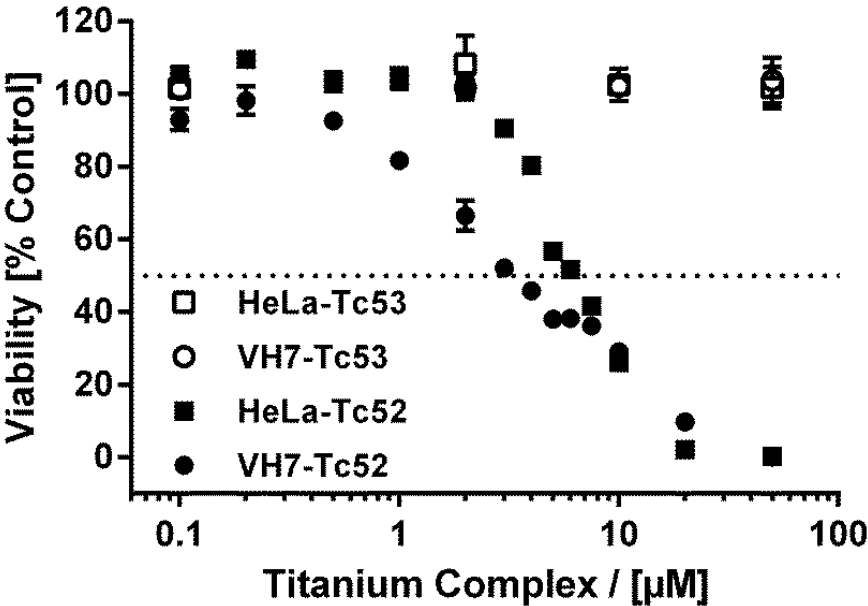

Supplement: Additional file 2: — Cell viability assay with HeLa tumor cells (squares) and VH7 normal fibroblasts (circles). Cells were exposed to increasing concentrations of Tc52 (filled) or Tc53 (open) titanium(IV)salan compounds solubilized in DMSO and incubated for 48 h. Subsequently, medium was replaced by fresh medium containing 9 μg/ml resazurin and cells were further incubated. Viability is expressed as % of solvent control. Whereas Tc53 is non-toxic in the tested concentration range, Tc52 impairs viability in HeLa and VH7 cells with an EC20 of 3 μM and 1 μM, an EC50 of 6 μM and 3 μM, respectively, and an EC80 of about 10 μM for both. (PDF 1085 kb) [file 12885_2016_2538_MOESM2_ESM.pdf]

## Additional Figure 5

Representative cell-cycle profiles 24 h after combined toxin application

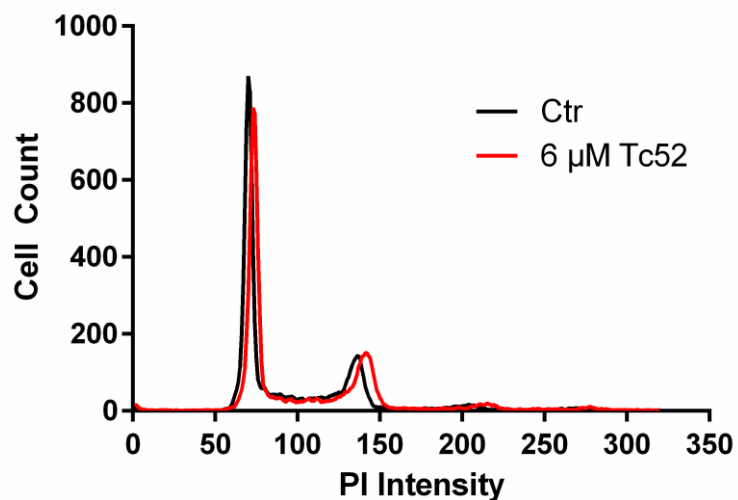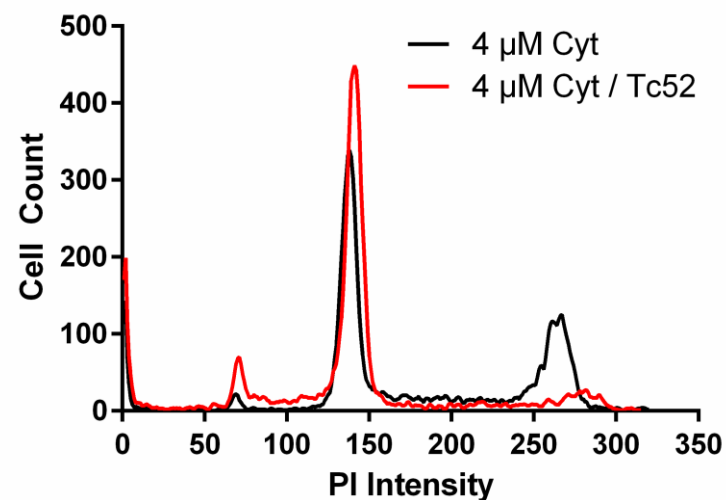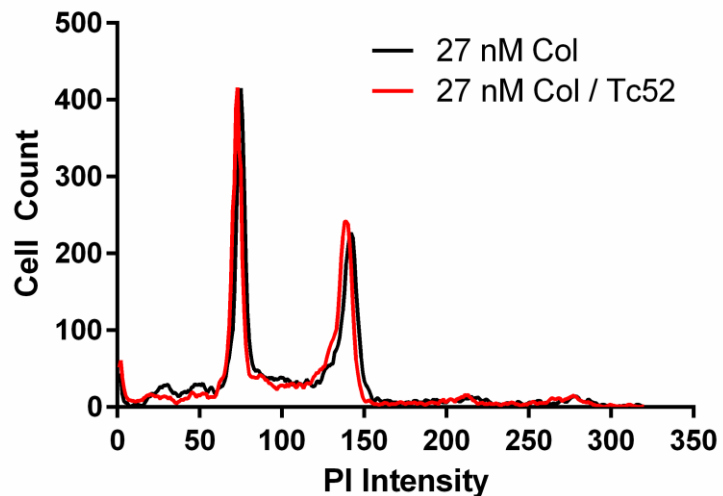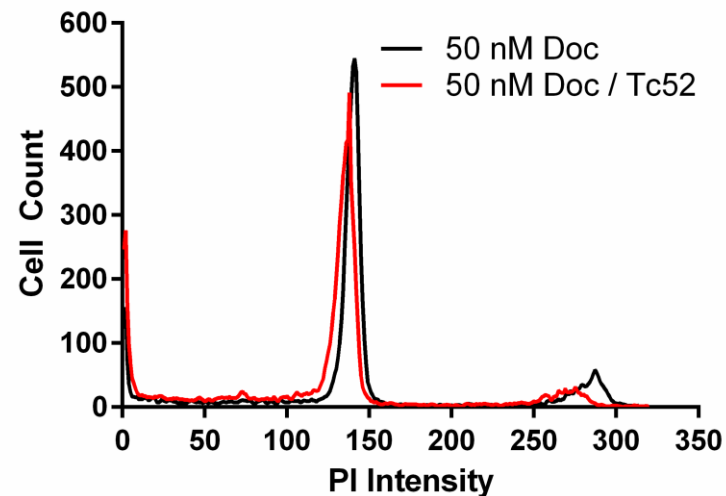

Supplement: Additional file 5: — Cell cycle distribution profile of HeLa cells after treatment with M-phase targeting toxins in combination with Tc52. Representative flow-cytometry histograms from data presented in Fig. 3. For better visibility, only one M-phase targeting toxin with or without 6 μM Tc52 is displayed in each panel. There is no significant difference in cell-cycle profiles of Tc52 or control samples except a small increase G2-phase in Tc52 treated cells (upper left panel). CytB-treated samples show a near-complete loss of G1-peak and a reduction in G2, concomitant with an increase in subG1 and the appearance of a substantial cell-fraction with a >4 N DNA content with a strong peak at about 8 N (compare black graphs from upper right and upper left panel). Combination with Tc52 reduces this 8 N peak and increases the number of cells in G2 and slightly the number of cells in G1 (upper right panel). Col-treatment induces a reduction in G1-phase (compare the black graphs from lower left and upper left panel) concomitant with an increase in G2 and subG1 (lower left panel). Combination with Tc52 does not change the cell-cycle profile significantly. 50 nM Doc induces a complete loss of cells from G1, concomitant with an increase in subG1 fraction, number of cells in G2 as wells as cells with a > 4 N DNA content (compare black graphs from lower right panel with upper left panel). Addition of Tc52 decreased the number of cells with a > 4 N DNA content (lower right panel). (PDF 1085 kb) [file 12885_2016_2538_MOESM5_ESM.pdf]

## Additional Figure 6

Representative cell-cycle profiles 30 h after 6 h Tc52 or Tc53 application

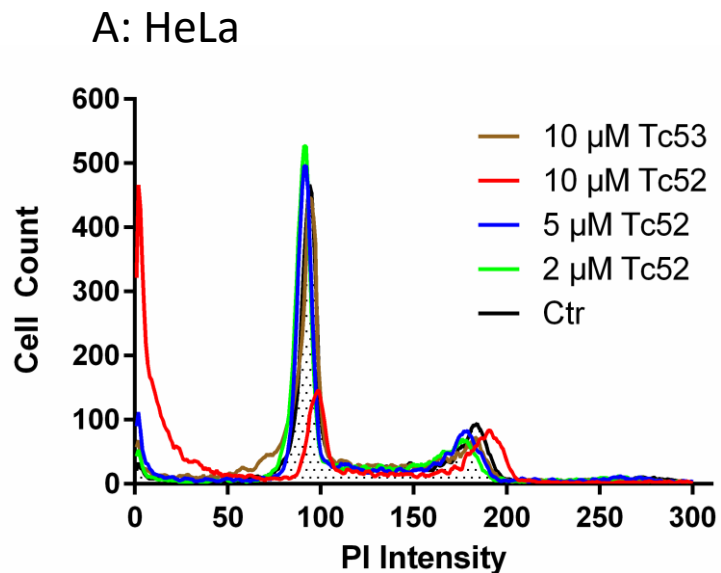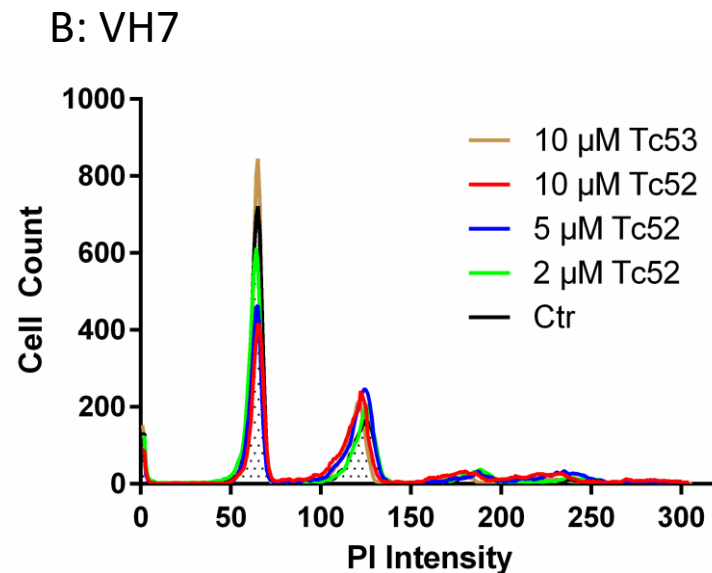

Supplement: Additional file 6: — Cell cycle distribution profile of cells after 6 h of treatment. Representative flow-cytometry histograms from data presented in Fig. 4. For better visibility, surface of the graph from Ctr sample (black line) is dotted. A: Cell-cycle distribution of HeLa cells after 6 h incubation with Titanium(IV)salan complexes Tc52 and Tc53 30 h after treatment start. Only 10 μM Tc52 shows significant impact on cell-cycle profile, i.e. severe reduction in G1 and increase of the subG1 fraction. B: Cell-cycle distribution of VH7 normal fibroblasts after 6 h incubation with Titanium(IV)salan complexes Tc52 and Tc53 30 h after treatment start. Samples exposed to 5 μM and 10 μM Tc52 display major reduction in number of cells in G1 concomitant with a mild increase in G2-phase. SubG1 fraction is not significantly elevated. (PDF 1086 kb) [file 12885_2016_2538_MOESM6_ESM.pdf]

## Additional Figure 7

### Cytosolic $\text{Ca}^{2+}$ measurement

A (HeLa)

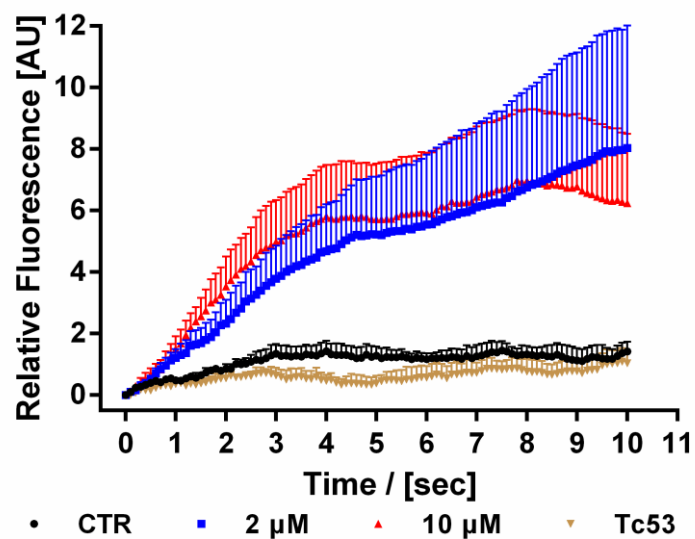

B (VH7)

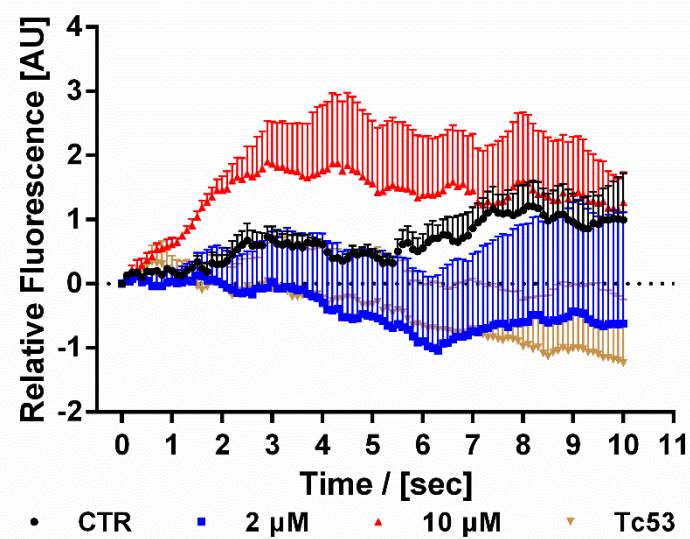

Supplement: Additional file 7: — Analysis of rapid free cytosolic Ca2 + -shifts in HeLa tumor cells and VH7 normal fibroblasts. Cells were processed as suggested by the manufacturer. Tc52 or Tc53 or solvent control (CTR) was administered to the cells and Ca2 + -dependent increase in fluorescence of Fluo4-NW-dye was measured. Significant increase in fluorescence is an indicator for cell death signaling. A: Increase in free cytosolic Ca2+ in HeLa cells. Both concentrations show significant and rapid increase in Ca2+ signal, reaching after 5 sec 4 and 5.5 RFU above CTR and after 10 sec 6.5 and 5 RFU above CTR, whereas control and Tc53 values stay low. B: No significant increase in free cytosolic Ca2+ in VH7 cells. Only the 10 μM Tc52 exposure samples display initially a mild increase (1 RFU above CTR), which is lost after 10 sec (0 RFU above CTR). As summary, rapid Ca2+ increase is indicative for cytotoxicity of Tc52 in HeLa cells, whereas its toxicity is negligible in VH7 normal fibroblasts, in line with other data. (PDF 1085 kb) [file 12885_2016_2538_MOESM7_ESM.pdf]

## Stress response after Tc53 application

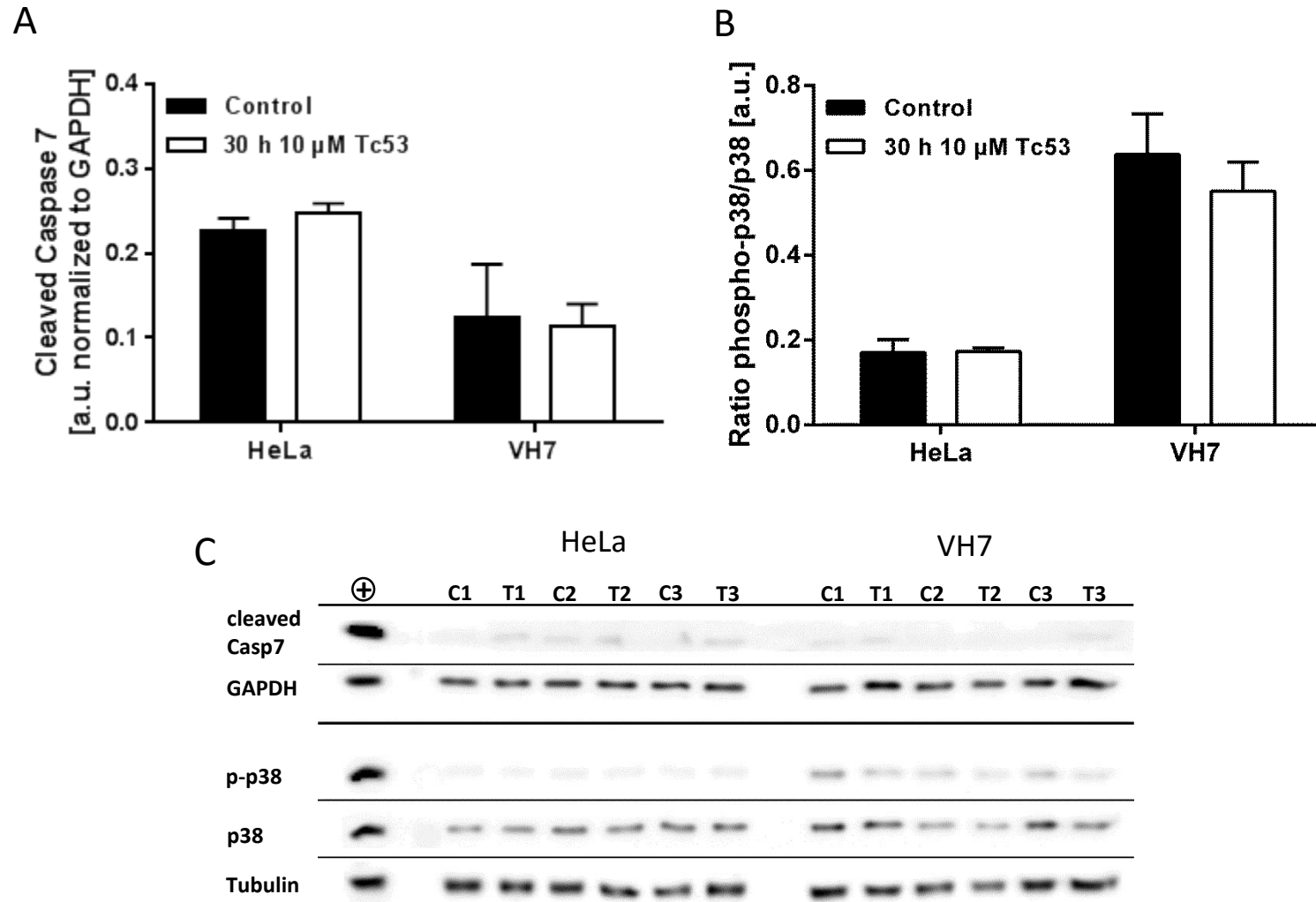

Supplement: Additional file 9: — Analysis of p38 kinase and caspase activation by Tc53 in HeLa and VH7 cells. Cells were exposed to 10 μM Tc53 or solvent for 30 h and subsequently lysed in Laemmli buffer. Western blot analysis was performed as described for Figs. 6 and 7. There is no evidence for activation of p38 in. HeLa or VH7 cells (A) or cleavage of caspase7 (B). Significance was tested using two-tailed T-test. Panel (C) depicts the respective western blot for cleaved caspase7 (cleaved Casp7), GAPDH, total p38 stresskinase (p38), phosphorylated p38 (p-p38) and αtubulin (Tubulin). +: positive control, C: solvent control samples, T: 10 μM Tc53 treated samples. Numbers indicate respective independent experiments. (PDF 1085 kb) [file 12885_2016_2538_MOESM9_ESM.pdf]

## Additional Figure 9

Viability after 48 h Tc52 or Tc53 application

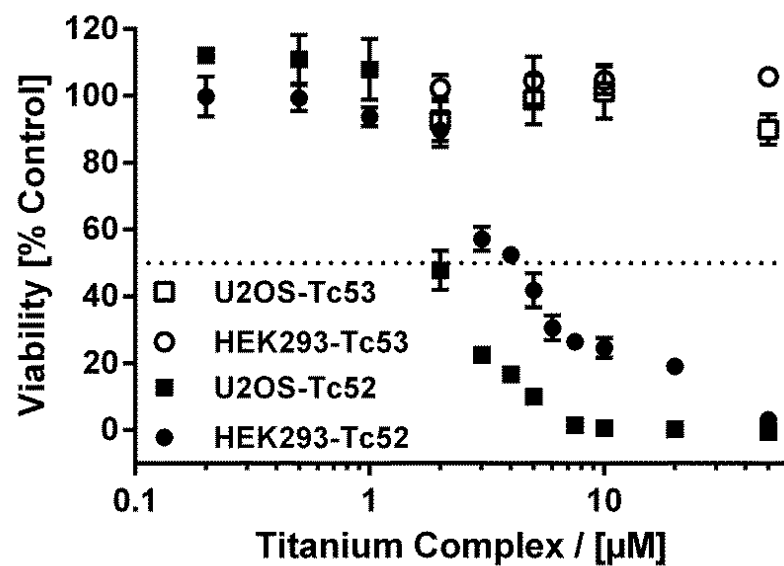

Supplement: Additional file 10: — Cell viability assay with U2OS tumor cells (squares) and low-passage HEK293 (circles). Cells were exposed to increasing concentrations of Tc52 (filled) or Tc53 (open) titanium(IV)salan compounds solubilized in DMSO and incubated for 48h. Subsequently, medium was replaced by fresh medium containing 9 μg/ml resazurin and cells were further incubated. Viability is expressed as % of solvent control. Whereas Tc53 is non-toxic in the tested concentration range, Tc52 impairs viability in U2OS and HEK293 cells with an EC20 of 1.5 μM and 2.5 μM, an EC50 of 2 μM and 4 μM, and an EC80 of about 4 μM and 20 μM, respectively. (PDF 1085 kb) [file 12885_2016_2538_MOESM10_ESM.pdf]
