# Supplementary material for: Differential cytotoxicity induced by the Titanium(IV)Salan complex Tc52 in G2-phase independent of DNA damage
Source: BMC Cancer. 2016 Jul 13;16:469. doi: 10.1186/s12885-016-2538-0 (PMC4944496; doi:10.1186/s12885-016-2538-0)

Additional Figure 3A

HeLa (H_2O_2)

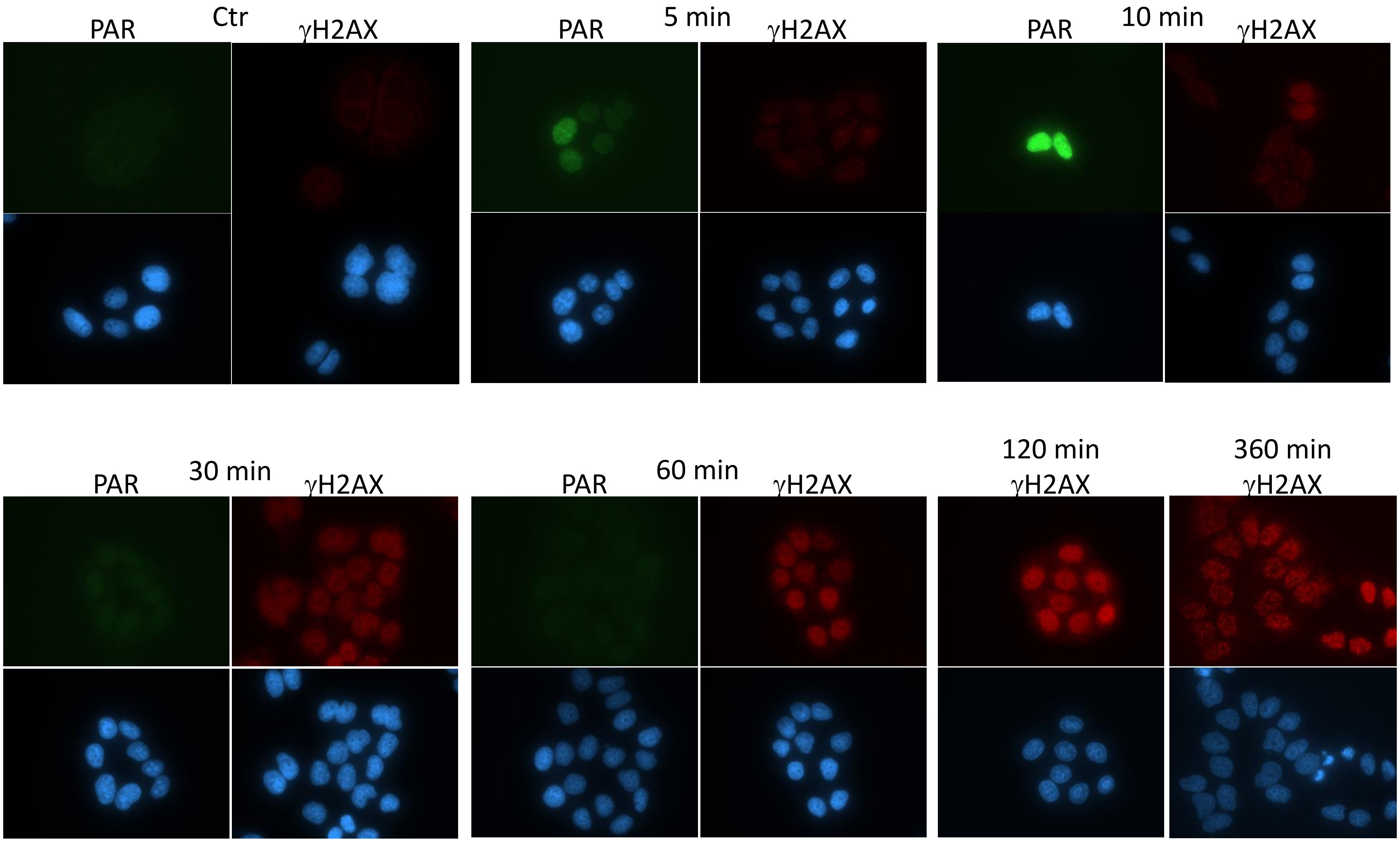

Additional Figure 3B

VH7 (H_2O_2)

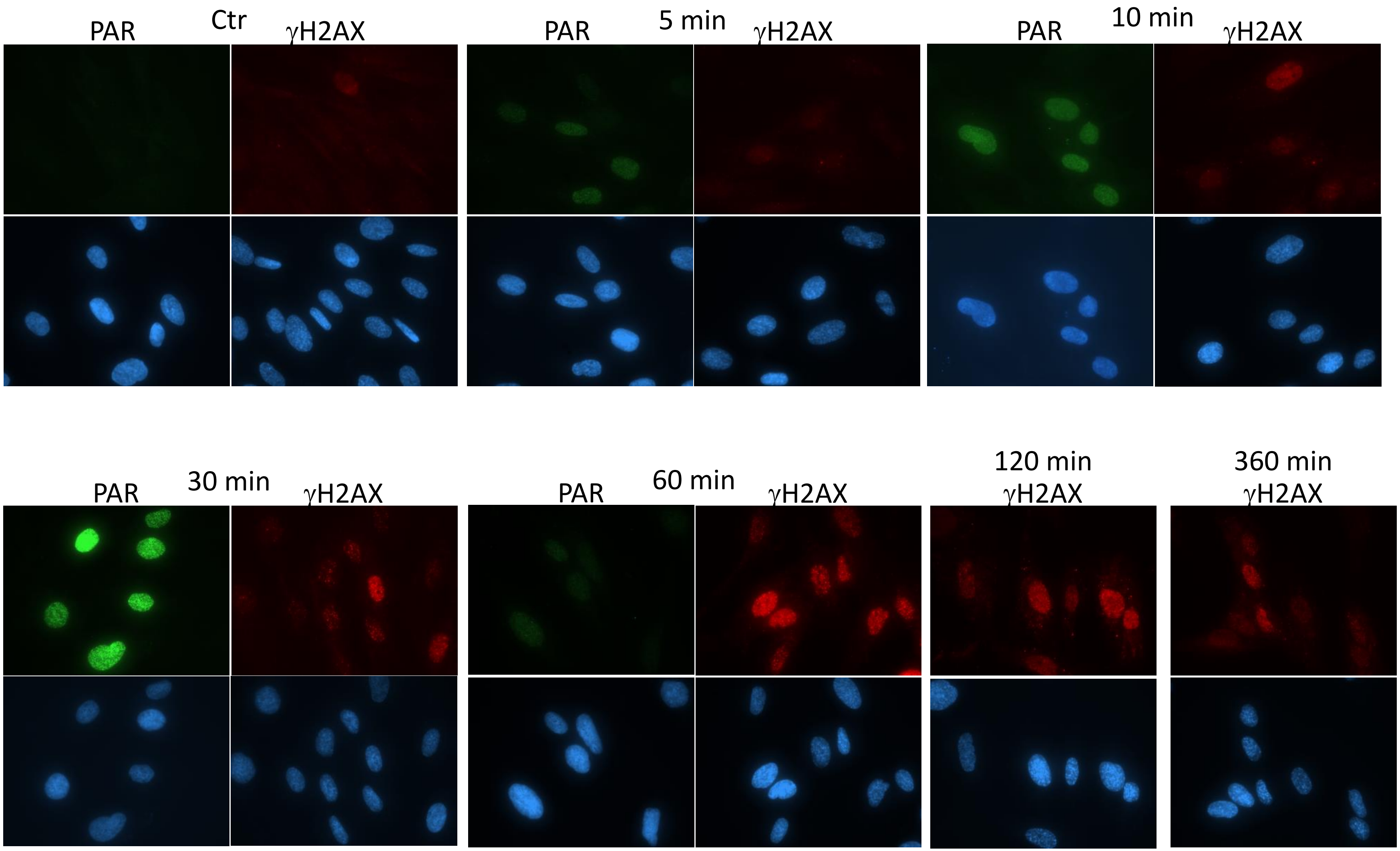

Additional Figure 3C

Time dependent γH2AX formation in HeLa cells

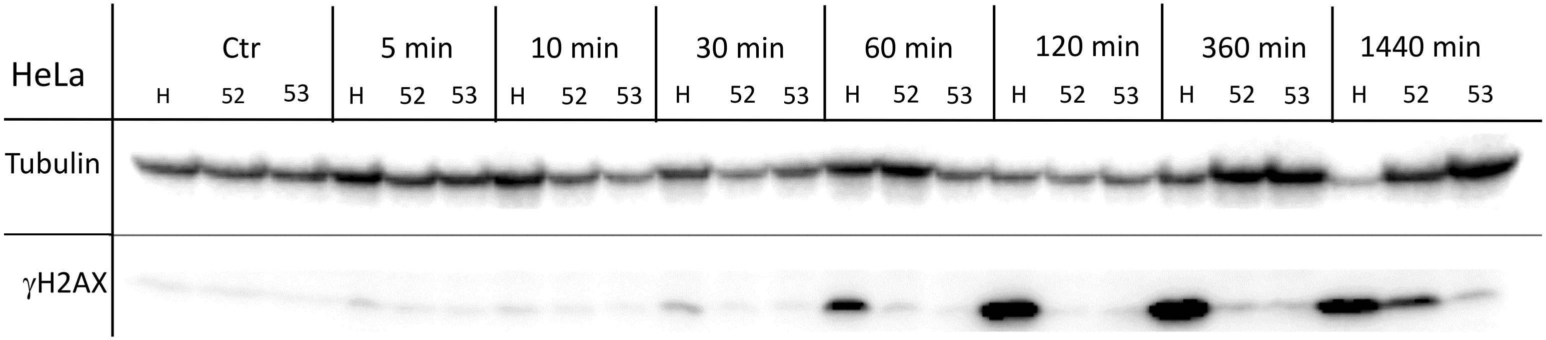

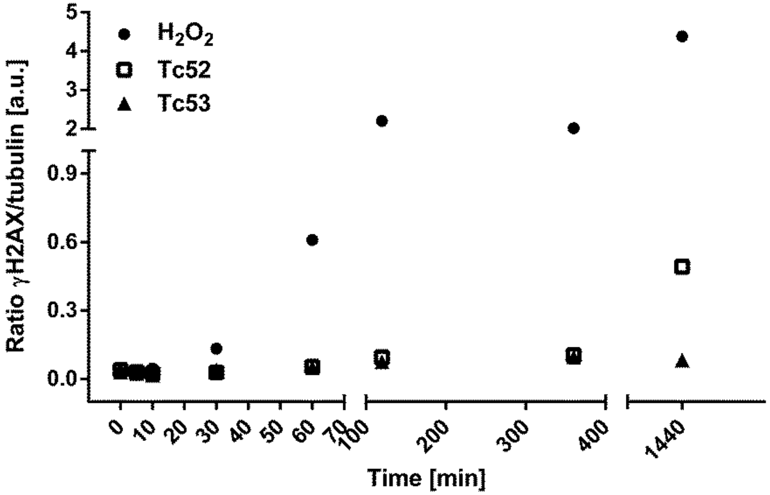

Supplement: Additional file 3: — Detection of DNA strand break markers poly(ADP-ribosyl)ation and γH2AX in HeLa and VH7. A: PAR can be weakly detected in HeLa cells about 5 min after application of 500 μM H2O2 in cell culture medium, peaking at 10 min, and signals disappear completely after 60 min. Later time points are not depicted. Phosphorylated H2AX appears after 10 min incubation as a pan-nuclear signal, with pronounced characteristic foci formation after 6 h. B: PAR can be weakly detected in VH7 cells about 5 min after application of 500 μM H2O2 in cell culture medium, peaking at 30 min and signals disappear completely after 60 min. Later time points are not depicted. Phosphorylated H2AX appears after 10 min incubation as a pan-nuclear signal, with less well pronounced characteristic foci formation after 60 min. C: H2AX and-tubulin detection in a time-course from 0 min (Ctr) to 1440 min (24 h) after application of 500 μM H2O2 (H), 10 μM Tc52 (52) or 10 μM Tc53 (53) to HeLa cells. Signal-ratio evaluation of γH2AX/tubulin is presented in the lower panel. γH2AX formation in H2O2 treated samples is evident after 30 min and increases over time. Only after 24 h of Tc52 treatment, a mild increase in γH2AX is detectable, concurrent with the onset of cell death (Fig. 1a). (PDF 1085 kb) [file 12885_2016_2538_MOESM3_ESM.pdf]
