# Supplementary material for: Differential cytotoxicity induced by the Titanium(IV)Salan complex Tc52 in G2-phase independent of DNA damage
Source: BMC Cancer. 2016 Jul 13;16:469. doi: 10.1186/s12885-016-2538-0 (PMC4944496; doi:10.1186/s12885-016-2538-0)

Additional Figure 4

Representative cell-cycle profiles after application of increasing doses of Tc52

A: 30 h

B: 48 h

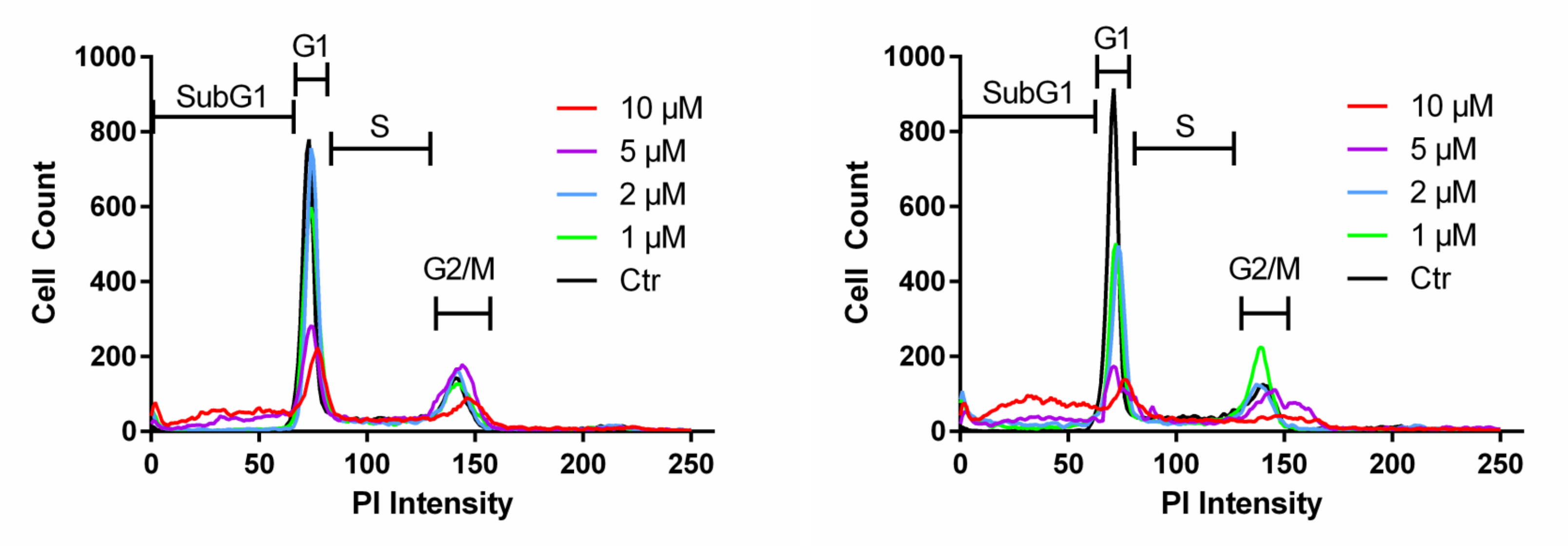

Supplement: Additional file 4: — Cell cycle distribution profile of HeLa cells after continuous Tc52 treatment. Representative histograms from data presented in Fig. 2. A: Cell-cycle distribution after 30 h of continuous treatment of cells with 1-10 μM Tc52 (concentration range showing an effect). Note the small (not significant) reduction in G1 with 2 μM Tc52 and the strong reduction with 5 μM and 10 μM Tc52 concomitant with a substantial increase in the subG1 fraction. The sample treated with 5 μM Tc52 displays in addition an increased number of cells in G2, whereas cells treated with 10 μM show a reduction in G2 phase. B: Cell-cycle distribution after 48 h of continuous treatment of cells with 1-10 μM Tc52 (concentration range showing an effect). Note the reduction in G1 with 1-2 μM Tc52 and the nearly complete loss in samples treated with 5 μM and 10 μM Tc52. SubG1 is increased in all samples and cells treated with 1 μM Tc52 display a significant increase in G2 phase. (PDF 1085 kb) [file 12885_2016_2538_MOESM4_ESM.pdf]
