# Supplementary material for: Differential cytotoxicity induced by the Titanium(IV)Salan complex Tc52 in G2-phase independent of DNA damage
Source: BMC Cancer. 2016 Jul 13;16:469. doi: 10.1186/s12885-016-2538-0 (PMC4944496; doi:10.1186/s12885-016-2538-0)
Supplement: Additional file 8: — Additional Methods. (PDF 144 kb) [file 12885_2016_2538_MOESM8_ESM.pdf]

Additional Methods

Free cytosolic Ca^{2+} assay

Analysis of free cytosolic Ca^{2+} was performed as described in [1, 2]. In brief, 20 000 cells/well seeded in 96well-plates (clear bottom/white walls, Corning) were washed twice with 49 parts of calcium-free HBSS (0.49 mM $MgCl_2$, 0.41 mM $MgSO_4$, 5.33 mM KCl, 0.44 mM KH_2PO4, 4.17 mM $NaHCO_3$, 137 mM NaCl, 0.34 mM Na_2HPO_4, 5.56 mM dextrose) supplemented with one part 1 M HEPES (pH 7.2) (Assay Buffer) containing 1 mM $CaCl_2$. 100 µl Fluo4-NW-dye-mix from Molecular Probes (Invitrogen) was added and incubated for 30 min at 37°C, followed by 30 min incubation in the dark at RT. Changes in relative fluorescence units (ΔRFU) from the Fluo4-NW-dye quantify alterations in free cytosolic Ca^{2+} concentrations (excitation/emission 485/535 nm; slits 10/15 nm) in LS55 spectrometer (Perkin-Elmer) after toxin treatment. Stock solutions of toxins were diluted in Assay Buffer to the desired concentration. Free cytosolic Ca^{2+} was monitored for the indicated time with a measure frequency of 0.1 sec. Significant increase in fluorescence indicates cell death signaling.

Additional Figures

Additional Figure 1

Structure of Tc52 and Tc53

Top structure highlights the identical backbone of Tc52 and Tc53. R (circled in red) indicates the two different side-chains of Tc52 (methyl-group) and Tc53 (tert-butyl-group), respectively. Depicted below are the separate structures for Tc52 and Tc53.

Additional Figure 2

Cell viability assay with HeLa tumor cells (squares) and VH7 normal fibroblasts (circles)

Cells were exposed to increasing concentrations of Tc52 (filled) or Tc53 (open) titanium(IV)salan compounds solubilized in DMSO and incubated for 48 h. Subsequently, medium was replaced by fresh medium containing 9 µg/ml resazurin and cells were further incubated. Viability is expressed as % of solvent control. Whereas Tc53 is non-toxic in the tested concentration range, Tc52 impairs viability in HeLa and VH7 cells with an EC_{20} of 3 µM and 1 µM, an EC_{50} of 6 µM and 3 µM, respectively, and an EC_{80} of about 10 µM for both.

Additional Figure 3

Detection of DNA strand break markers poly(ADP-ribosyl)ation and γH2AX in HeLa and VH7

A: PAR can be weakly detected in HeLa cells about 5 min after application of 500 µM H_2O_2 in cell culture medium, peaking at 10 min, and signals disappear completely after 60 min. Later time points are not depicted. Phosphorylated H2AX appears after 10 min incubation as a pan-nuclear signal, with pronounced characteristic foci formation after 6 h.

B: PAR can be weakly detected in VH7 cells about 5 min after application of 500 µM H_2O_2 in cell culture medium, peaking at 30 min and signals disappear completely after 60 min. Later time points are not depicted. Phosphorylated H2AX appears after 10 min incubation as a pan-nuclear signal, with less well pronounced characteristic foci formation after 60 min.

C: γH2AX and α-tubulin detection in a time-course from 0 min (Ctr) to 1440 min (24 h) after application of 500 µM H_2O_2 (H), 10 µM Tc52 (52) or 10 µM Tc53 (53) to HeLa cells. Signal-ratio evaluation of γH2AX/tubulin is presented in the lower panel. γH2AX formation in H_2O_2 treated samples is evident after 30 min and increases over time. Only after 24 h of Tc52 treatment, a mild increase in γH2AX is detectable, concurrent with the onset of cell death (Fig.1A).

Additional Figure 4

Cell cycle distribution profile of HeLa cells after continuous Tc52 treatment

Representative histograms from data presented in Figure 2.

A: Cell-cycle distribution after 30 h of continuous treatment of cells with 1-10 µM Tc52 (concentration range showing an effect). Note the small (not significant) reduction in G1 with 2 µM Tc52 and the strong reduction with 5 µM and 10 µM Tc52 concomitant with a substantial increase in the subG1 fraction. The sample treated with 5 µM Tc52 displays in addition an increased number of cells in G2, whereas cells treated with 10 µM show a reduction in G2 phase.

B: Cell-cycle distribution after 48 h of continuous treatment of cells with 1-10 µM Tc52 (concentration range showing an effect). Note the reduction in G1 with 1-2 µM Tc52 and the nearly complete loss in samples treated with 5 µM and 10 µM Tc52. SubG1 is increased in all samples and cells treated with 1 µM Tc52 display a significant increase in G2 phase.

Additional Figure 5

Cell cycle distribution profile of HeLa cells after treatment with M-phase targeting toxins in combination with Tc52

Representative flow-cytometry histograms from data presented in Figure 3. For better visibility, only one M-phase targeting toxin with or without 6 µM Tc52 is displayed in each panel.

There is no significant difference in cell-cycle profiles of Tc52 or control samples except a small increase G2-phase in Tc52 treated cells (upper left panel).

CytB-treated samples show a near-complete loss of G1-peak and a reduction in G2, concomitant with an increase in subG1 and the appearance of a substantial cell-fraction with a >4 N DNA content with a strong peak at about 8 N (compare black graphs from upper right and upper left panel). Combination with Tc52 reduces this 8 N peak and increases the number of cells in G2 and slightly the number of cells in G1 (upper right panel).

Col-treatment induces a reduction in G1-phase (compare the black graphs from lower left and upper left panel) concomitant with an increase in G2 and subG1 (lower left panel). Combination with Tc52 does not change the cell-cycle profile significantly.

50 nM Doc induces a complete loss of cells from G1, concomitant with an increase in subG1 fraction, number of cells in G2 as wells as cells with a > 4 N DNA content (compare black graphs from lower right panel with upper left panel). Addition of Tc52 decreased the number of cells with a > 4 N DNA content (lower right panel).

<u>Additional Figure 6</u>

Cell cycle distribution profile of cells after 6 h of treatment

Representative flow-cytometry histograms from data presented in Figure 4. For better visibility, surface of the graph from Ctr sample (black line) is dotted.

A: Cell-cycle distribution of HeLa cells after 6 h incubation with Titanium(IV)salan complexes Tc52 and Tc53 30 h after treatment start. Only 10 µM Tc52 shows significant impact on cell-cycle profile, i.e. severe reduction in G1 and increase of the subG1 fraction.

B: Cell-cycle distribution of VH7 normal fibroblasts after 6 h incubation with Titanium(IV)salan complexes Tc52 and Tc53 30 h after treatment start. Samples exposed to 5 µM and 10 µM Tc52 display major reduction in number of cells in G1 concomitant with a mild increase in G2-phase. SubG1 fraction is not significantly elevated.

Additional Figure 7

Analysis of rapid free cytosolic Ca^{2+}-shifts in HeLa tumor cells and VH7 normal fibroblasts

Cells were processed as suggested by the manufacturer. Tc52 or Tc53 or solvent control (CTR) was administered to the cells and Ca^{2+}-dependent increase in fluorescence of Fluo4-NW-dye was measured. Significant increase in fluorescence is an indicator for cell death signaling [1].

A: Increase in free cytosolic Ca^{2+} in HeLa cells. Both concentrations show significant and rapid increase in Ca^{2+} signal, reaching after 5 sec 4 and 5.5 RFU above CTR and after 10 sec 6.5 and 5 RFU above CTR, whereas control and Tc53 values stay low.

B: No significant increase in free cytosolic Ca^{2+} in VH7 cells. Only the 10 µM Tc52 exposure samples display initially a mild increase (1 RFU above CTR), which is lost after 10 sec (0 RFU above CTR).

As summary, rapid Ca^{2+} increase is indicative for cytotoxicity of Tc52 in HeLa cells, whereas its toxicity is negligible in VH7 normal fibroblasts, in line with other data.

Additional Figure 8

Analysis of p38 kinase and caspase activation by Tc53 in HeLa and VH7 cells

Cells were exposed to 10 µM Tc53 or solvent for 30 h and subsequently lysed in Laemmli buffer. Western blot analysis was performed as described for Figure 6 and 7. There is no evidence for activation of p38 in HeLa or VH7 cells (**A**) or cleavage of caspase7 (**B**). Significance was tested using two-tailed T-test. Panel (**C**) depicts the respective western blot for cleaved caspase7 (cleaved Casp7), GAPDH, total p38 stress-kinase (p38), phosphorylated p38 (p-p38) and α-tubulin (Tubulin). +: positive control, C: solvent control samples, T: 10 µM Tc53 treated samples. Numbers indicate respective independent experiments.

Additional Figure 9

Cell viability assay with U2OS tumor cells (squares) and low-passage HEK293 (circles)

Cells were exposed to increasing concentrations of Tc52 (filled) or Tc53 (open) titanium(IV)salan compounds solubilized in DMSO and incubated for 48h. Subsequently, medium was replaced by fresh

medium containing 9 µg/ml resazurin and cells were further incubated. Viability is expressed as % of solvent control. Whereas Tc53 is non-toxic in the tested concentration range, Tc52 impairs viability in U2OS and HEK293 cells with an EC_{20} of 1.5 µM and 2.5 µM, an EC_{50} of 2 µM and 4 µM, and an EC_{80} of about 4 µM and 20 µM, respectively.

Additional References

1. Wyrsch P, Blenn C, Pesch T, Beneke S, Althaus FR: Cytosolic Ca2+ shifts as early markers of cytotoxicity. *Cell Commun Signal* 2013, 11(1):11.
2. Blenn C, Wyrsch P, Bader J, Bollhalder M, Althaus FR: Poly(ADP-ribose)glycohydrolase is an upstream regulator of Ca2+ fluxes in oxidative cell death. *Cell Mol Life Sci* 2011, 68(8):1455-1466.
